# Actin-binding protein coronin 1A controls osteoclastic bone resorption by regulating lysosomal secretion of cathepsin K

**DOI:** 10.1038/srep41710

**Published:** 2017-03-16

**Authors:** Saori Ohmae, Naruto Noma, Masayasu Toyomoto, Masahiro Shinohara, Masatoshi Takeiri, Hiroaki Fuji, Kenji Takemoto, Keiko Iwaisako, Tomoko Fujita, Norihiko Takeda, Makoto Kawatani, Mineyoshi Aoyama, Masatoshi Hagiwara, Yasushi Ishihama, Masataka Asagiri

**Affiliations:** 1 Innovation Center for Immunoregulation and Therapeutics, Graduate School of Medicine, Kyoto University, Sakyo-ku, Kyoto, Japan; 2 Department of Bioimaging and Cell Signaling, Graduate School of Biostudies, Kyoto University, Sakyo-ku, Kyoto, Japan; 3 Department of Anatomy and Developmental Biology, Graduate School of Medicine, Kyoto University, Sakyo-ku, Kyoto, Japan; 4 Department of Systems BioMedicine, Tokyo Medical and Dental University, Bunkyo-ku, Tokyo, Japan; 5 PRESTO, Japan Science and Technology Agency, Kawaguchi, Saitama, Japan; 6 Division of Hepato-Biliary-Pancreatic Surgery and Transplantation, Department of Surgery, Graduate School of Medicine, Kyoto University, Sakyo-ku, Kyoto, Japan; 7 Department of Target Therapy Oncology, Graduate School of Medicine, Kyoto University, Sakyo-ku, Kyoto, Japan; 8 Department of Cardiovascular Medicine, Graduate School of Medicine, The University of Tokyo, Bunkyo-ku, Tokyo, Japan; 9 Chemical Biology Research Group, RIKEN CSRS, Wako, Saitama, Japan; 10 Department of Pathobiology, Graduate School of Pharmaceutical Sciences, Nagoya City University, Nagoya, Japan; 11 Department of Molecular and Cellular BioAnalysis, Graduate School of Pharmaceutical Sciences, Kyoto University, Sakyo-ku, Kyoto, Japan

## Abstract

Osteoclasts degrade bone matrix proteins via the secretion of lysosomal enzymes. However, the precise mechanisms by which lysosomal components are transported and fused to the bone-apposed plasma membrane, termed ruffled border membrane, remain elusive. Here, we identified coronin 1A as a negative regulator of exocytotic release of cathepsin K, one of the most important bone-degrading enzymes in osteoclasts. The modulation of coronin 1A expression did not alter osteoclast differentiation and extracellular acidification, but strongly affected the secretion of cathepsin K and osteoclast bone-resorption activity, suggesting the coronin 1A-mediated regulation of lysosomal trafficking and protease exocytosis. Further analyses suggested that coronin 1A prevented the lipidation-mediated sorting of the autophagy-related protein LC3 to the ruffled border and attenuated lysosome–plasma membrane fusion. In this process, the interactions between coronin 1A and actin were crucial. Collectively, our findings indicate that coronin 1A is a pivotal component that regulates lysosomal fusion and the secretion pathway in osteoclast-lineage cells and may provide a novel therapeutic target for bone diseases.

Osteoclasts differentiate from cells of the monocyte/macrophage lineage by the fusion of precursor cells into multinucleated cells in response to macrophage colony-stimulating factor (M-CSF) and receptor activator of nuclear factor kappa-B ligand (RANKL)^1^,^2^. RANKL-induced activation of the nuclear factor kappa-B (NF-κB) and mitogen-activated protein kinase (MAPK) signalling pathways result in the increase in nuclear factor of activated T cells cytoplasmic 1 (NFATc1) and the expression of its target genes, including those encoding cathepsin K, ATPase H^+^ transporting V0 subunit d2 (Atp6v0d2) and tartrate-resistant acid phosphatase (TRAP)^3^,^4^. Osteoclasts resorb bones by attaching to the bone surface and forming a ruffled border, which is a part of the plasma membrane surrounded by an actin ring tightly adhered to the bone matrix; and by secreting lysosomal proteases such as cathepsin K from the ruffled border membranes into the acidic extracellular space^5^,^6^,^7^. In fact, delivery of lysosomal vesicles containing bone-degrading proteases to the ruffled border and the related exocytotic events are necessary for precisely controlled bone resorption^8^,^9^,^10^. Because an imbalance of bone homeostasis such as excessive or defective bone resorption causes various skeletal disorders^11^, there is a tremendous need for improved understanding of the mechanisms of bone resorption. Previous studies showed that the formation of a ruffled border and/or the transport of secretary lysosome requires synaptotagmin VII, Rab7, Rab27A and protein-kinase C (PKC) δ^9^,^12^,^13^,^14^. However, the detailed mechanisms of delivery or fusion of lysosomal vesicles to the ruffled border membrane in osteoclasts remain poorly understood.

Similarly to the lysosomal fusion with ruffled border membranes in osteoclasts, autophagy is a system that requires dynamic membrane trafficking and fusion with the lysosome. Autophagy, which is a process of self-degradation of cellular components via lysosomal fusion, contributes to the adaptation to a nutritionally deficient state and maintenance of cellular homeostasis through removing damaged organelles, invasive pathogens and aggregated proteins^15^,^16^,^17^. However, recent studies suggested that autophagy also participates in osteoclast differentiation and function^18^,^19^. Deletion of autophagy-related (Atg) 5 or Atg 7 in cells of the monocyte/macrophage lineage impairs the proper assembly of the ruffled border and reduces localization of the autophagic protein LC3 within actin rings, resulting in lower resorption activity *in vitro* and slightly increased bone volume *in vivo*^20^,^21^. These reports suggest that autophagic proteins are involved in proper osteoclast formation and osteoclastic bone resorption.

Coronin 1A is a member of the coronin family of actin-associated proteins and is highly expressed in hematopoietic cells^22^,^23^,^24^. This protein contains 461 amino acids and is comprised of the following three functional domains: an amino-terminal tryptophan-aspartic acid (WD) repeat-containing region followed by a unique region and a carboxy-terminal coiled-coil domain^22^,^23^. Because coronin 1A interacts with actin, it has been assumed to be involved in cytoskeletal regulation, however, coronin 1A-deficient macrophages exhibit intact cell motility and phagocytosis^22^,^23^. On the other hand, coronin 1A has been identified as an inhibitor of the lysosomal degradation pathway by preventing the fusion of endosomes/phagosomes with lysosomes^25^,^26^,^27^,^28^. Furthermore, coronin 1A is important for calcium mobilization and calcineurin activation^25^,^27^,^29^. Of note, both calcium–calcineurin signalling and lysosomal fusion are necessary for osteoclast differentiation and function, respectively; the calcium–calcineurin signalling-induced activation of NFATc1 is essential for osteoclasts differentiation, and the fusion of lysosomal vesicles to ruffled border plasma membrane is important for the release of bone-degrading enzymes that function in osteoclastic bone resorption. However, the role of coronin 1A in osteoclasts is unknown. In the present study, we investigated the involvement of coronin 1A in osteoclast differentiation and function and revealed that the modulation of coronin 1A expression levels alteres the activity of osteoclastic bone resorption without affecting osteoclastogenesis and that coronin 1A inhibits lysosome fusion to plasma membrane of the ruffled border and cathepsin K secretion.

## Results

### Expression patterns of coronin 1A and its localization during osteoclast differentiation

**Figure 1:**
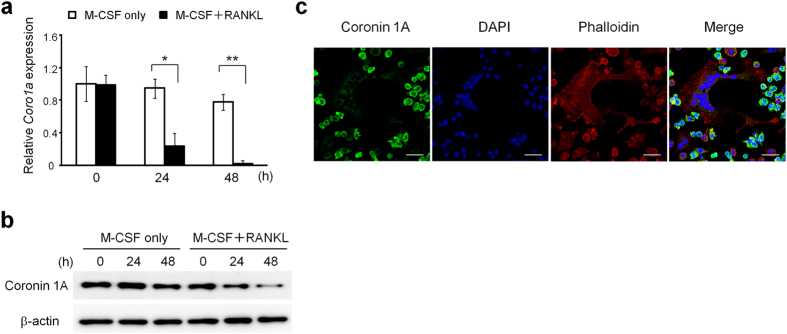
Coronin 1A was decreased during osteoclast differentiation. (**a**,**b**) The expression of coronin 1A during osteoclastogenesis. The bone marrow macrophages (BMMs) were cultured in the presence of macrophage colony-stimulating factor (M-CSF) (10 ng/mL) and receptor activator of nuclear factor kappa-B ligand (RANKL) (50 ng/mL) for the indicated times, and analysed *Coro1a* mRNA (**a**) and coronin 1A protein level (**b**). Unprocessed original scans of blots are shown in Supplementary Fig. 10. *P < 0.05, **P < 0.01, (Student’s t-test). Data on *Coro1a* mRNA expression are means ± standard deviation (SD) of three independent experiments. (**c**) The intracellular localization of coronin 1A in osteoclasts. The osteoclasts were fixed, stained with anti-coronin 1A antibody, DAPI and phalloidin, and observed by confocal microscopy. Scale bars, 20 μm. Data are representative of three experiments.

We hypothesized that the actin-binding protein coronin 1A might play a crucial role in osteoclast differentiation and/or function. Therefore, we first analysed the expression and localization of coronin 1A during osteoclast differentiation. Coronin 1A was highly expressed in osteoclast precursor cells (bone marrow macrophages; BMMs) and was decreased during osteoclastogenesis in both mRNA and protein levels (Fig. 1a,b). Coronin 1A expression was decreased in BMMs from 24 h after RANKL stimulation and continued to decrease until 48 h. Coronin was first identified in *Dictyostelium* and was characterized as an actin-binding protein. Consistent with the previous reports^22^,^24^,^30^, coronin 1A was distributed near the plasma membrane and co-localized to F-actin (Fig. 1c). Of note, coronin 1A was detected in osteoclast precursor cells (Fig. 1c: small cells), but to a lesser extent in mature osteoclasts (Fig. 1c: multinucleated cells [MNCs, >3 nuclei]), confirming that coronin 1A is reduced during osteoclastogenesis.

### Role of coronin 1A in RANKL-dependent osteoclastogenesis

**Figure 2:**
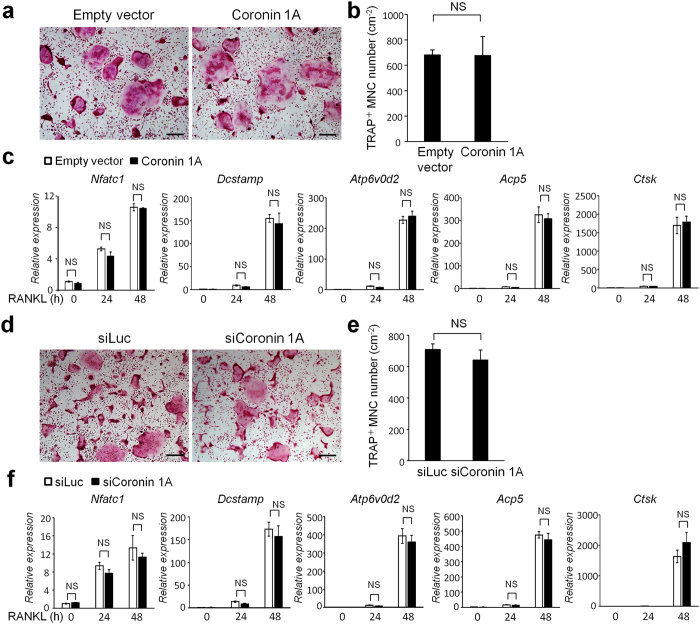
Coronin 1A was dispensable for osteoclastogenesis. (**a**–**c**) BMMs were transduced with CSII-CMV-MCS-IRES2-Venus (empty vector) lentivirus or with lentivirus expressing coronin 1A, and then were cultured with M-CSF (10 ng/mL) and RANKL (50 ng/mL) to differentiate into osteoclasts. (**a**,**b**) Cultured cells were fixed and stained for tartrate-resistant acid phosphatase (TRAP). The numbers of TRAP-positive multinucleated cells (MNCs) (>3 nuclei) were counted. Data are means ± SD of three independent experiments. Scale bars, 200 μm. (Student’s t-test), NS: not-statistically significant. (**c**) Expression of osteoclast marker genes, *Nfatc1, Dcstamp, Atp6v0d2, Acp5 and Ctsk* during osteoclastogenesis. Total RNA was extracted from the cultured cells at the indicated time points and subjected to real-time PCR. (Student’s t-test), NS: not-statistically significant. (**d**–**f**) BMMs were transfected with luciferase- or coronin 1A-specific siRNA, and then were incubated with M-CSF and RANKL. (**d**,**e**), Osteoclast differentiation of the control and coronin 1A knockdown cells. The numbers of TRAP-positive MNCs (>3 nuclei) were counted. Data are means ± SD of three independent experiments. Scale bars, 200 μm. (Student’s t-test), NS: not-statistically significant. (**f**) Expression of osteoclast marker genes during osteoclast differentiation. Data are representative of three experiments. (Student’s t-test), NS: not-statistically significant.

We next investigated whether coronin 1A is required for RANKL-induced osteoclastogenesis. Primary BMMs infected with empty lentiviral vector or coronin 1A-expressing lentivirus were cultured with M-CSF and RANKL (Supplementary Fig. 1a,b), following which they were stained for TRAP, a marker of osteoclasts. Despite the importance of coronin 1A in calcium–calcineurin signalling^25^,^27^,^29^, which had been shown to be important for osteoclastogenic NFATc1 autoamplification^3^,^4^,^31^, the enforced coronin 1A expression did not affect RANKL-induced osteoclast differentiation (Fig. 2a,b) and NFATc1 induction (Fig. 2c) as well as precursor cell proliferation or survival (Supplementary Fig. 2a,b). Similar to the effects of overexpression of coronin 1A, its knockdown in BMMs did not affect osteoclastogenesis (Fig. 2d–f, Supplementary Fig. 1c) and precursor cell proliferation or survival (Supplementary Fig. 2c,d). Subsequently, we examined the role of coronin 1A in RANKL-dependent signalling pathways, including extracellular signal-regulated kinase (ERK), p38, c-Jun N-terminal kinases (JNK), NF-κB and v-akt murine thymoma viral oncogene (AKT), which are activated via the RANKL-TNF receptor-associated factor 6 (TRAF6)-dependent pathway^32^. As shown in Supplementary Fig. 3a and b, neither coronin 1A overexpression nor knockdown affected RANKL-dependent NF-κB, MAPK and AKT activation. In addition, mRNA expression levels of the osteoclastogenic markers *Nfatc1, Dcstamp*, ATPase H^+^ transporting V0 subunit d2 (*Atp6v0d2*), *Acp5* and *Ctsk* were not significantly affected by coronin 1A overexpression or its knockdown (Fig. 2c,f). These results indicated that the role of coronin 1A in osteoclast differentiation is minute.

### Osteoclastic bone resorption activity regulated by coronin 1A

**Figure 3:**
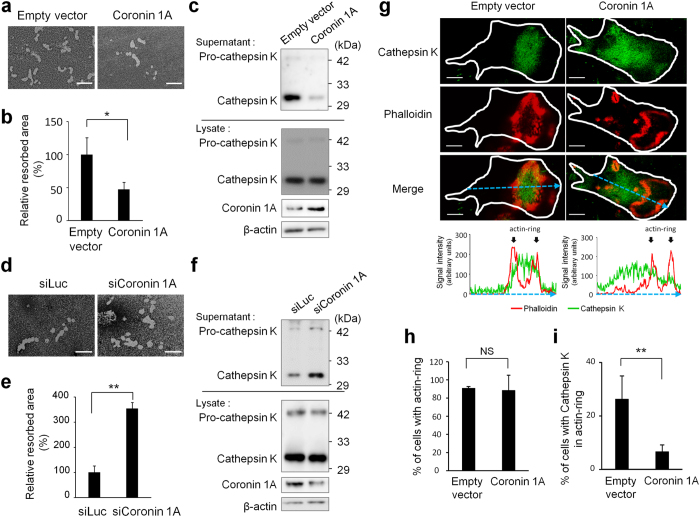
Coronin 1A inhibited bone resorption in osteoclasts. (**a**–**c**,**g**–**j**) BMMs were transduced with empty vector- (control) or coronin 1A-expressing lentivirus, and then were cultured with M-CSF (10 ng/mL) and RANKL (50 ng/mL) to differentiate into osteoclasts. (**a**,**b**) Osteoclasts were removed from the bone biomimetic synthetic surface plate and resorption pit areas were visualized by von Kossa staining. The bone resorption areas were analysed by using the MetaMorph software. Data are means ± SD of three independent experiments. *P < 0.05 for the indicated comparisons. Scale bars, 50 μm. (**c**) The amount of cathepsin K in cell lysates and supernatants. Cell lysates and supernatants were analysed by immunoblotting. Unprocessed original scans of blots are shown in Supplementary Fig. 10. (**d**–**f**) BMMs were transfected with luciferase- or coronin 1A-specific siRNA, and then were incubated with M-CSF and RANKL. (**d**,**e**) Bone resorption activity of control and coronin 1A knockdown cells. The resorption areas were analysed by using the MetaMorph software. Data are means ± SD of three independent experiments. **P < 0.01 for the indicated comparisons. Scale bars, 50 μm. (**f**) The amount of cathepsin K in cell lysates and supernatants. Unprocessed original scans of blots are shown in Supplementary Fig. 10. (**g**) Representative confocal images of control- and coronin 1A-overexpressing osteoclasts; actin, red; cathepsin K, green; cell perimeters, white. Scale bars, 20 μm. Graph indicates the intensity profile of the blue dotted line in each image. The blue dotted line passes through the centroid positions of each cell and actin-ring. (**h**) Percentage of control- or coronin 1A-overexpressing osteoclasts with actin ring; data are presented as mean ± SD, n = 35 in each group. (Student’s t-test). NS: not-statistically significant. (**i**,**j**) Percentage of cells with cathepsin K or lysosomal-associated membrane protein 1 (LAMP1) localized in actin-ring of control- or coronin 1A-overexpressing osteoclasts; data are presented as mean ± SD, n = 35 in each group. *P < 0.05, **P < 0.01, (Student’s t-test). Data are representative of three experiments.

Bone resorption requires lysosomal trafficking to and fusion with ruffled border^8^,^9^,^10^. Since coronin 1A has been identified to inhibit lysosomal fusion^25^,^26^,^27^,^28^, we assessed whether coronin 1A is involved in osteoclastic bone resorption activity. When osteoclasts were cultured on the bone biomimetic synthetic surface, resorption pits formed by coronin 1A-overexpressing osteoclasts were significantly smaller than those formed by control cells (Fig. 3a,b). Previous studies showed that cathepsin K is a key player in bone resorption via degradation of bone-matrix proteins, type I and type II collagen^33^,^34^,^35^,^36^. Thus, we focused on cathepsin K and assessed the amount of cathepsin K in whole cell lysates and culture supernatants by immunoblotting. We found that the amount of cathepsin K in the culture supernatants of coronin 1A-overexpressing osteoclasts was significantly smaller than that of control cells, whereas the total expression levels of cathepsin K in whole cell lysates did not differ between coronin 1A-overexpressing cells and control cells (Fig. 3c). In line with these results, the bone resorption area and the amount of cathepsin K in the culture supernatants tended to increase in osteoclasts transfected with coronin 1A specific small interfering (si)RNA, without any changes in the expression of cathepsin K (Fig. 3d–f), suggesting that coronin 1A inhibits cathepsin K secretion from osteoclasts. Although cytokines are also contained in small secretary vesicles and are transported to the plasma membrane and released out into the extracellular space^37^, the secretion of proinflammatory cytokines, such as tumor necrosis factor (TNF), interleukin 6 (IL-6) and IL-12/IL–23p40 from BMMs was not affected by coronin 1A overexpression or its knockdown (Supplementary Fig. 4a,b). The extracellular acidification of osteoclast resorption lacunae, which is mediated by vacuolar H^+^ -ATPases (V-ATPases), is a prerequisite for efficient degradation of bone matrix proteins by cathepsin K^10^,^38^,^39^. Thus, we investigated whether coronin 1A is involved in the extracellular acidification of osteoclasts. Using Acridine orange staining assay, which has been widely used to reveal the extracellular acidification and/or proton-pump activity in osteoclasts^40^,^41^,^42^,^43^, we found that the extracellular acidification was not affected by the coronin 1A-overexpression or -knockdown compared with control cells (Supplementary Fig. 5a,b), suggesting that coronin 1A regulation of the osteoclast proton secretion is not likely. These results together suggested that coronin 1A plays an important and specific role in the secretion of lysosomal enzymes such as cathepsin K but not in the secretion of cytokines and osteoclast extracellular acidification.

### Coronin 1A inhibits the localization of cathepsin K at ruffled border

We then focused on the influence of coronin 1A overexpression on spatiotemporal regulation of cathepsin K. We first investigated the actin-ring formation during osteoclast differentiation. Coronin 1A-overexpressing osteoclasts normally formed actin rings in response to RANKL (Fig. 3g,h), suggesting that cytoskeleton organization and the signals that activate this process are intact. However, the amount of cathepsin K localized within actin rings was greatly reduced by coronin 1A overexpression (Fig. 3g,i), indicating that coronin 1A prevents the sorting and/or fusion of cathepsin K-containing lysosomes to ruffled border membrane surrounded by actin rings. To obtain a more direct evidence of the regulatory role of coronin 1A in the fusion of lysosomes with the plasma membrane of ruffled border, we employed widely used LAMP1 (a lysosome marker) staining to assess the lysosomal fusion and found that coronin 1A overexpression impaired RANKL-induced localization of LAMP1 within osteoclast actin rings, which normally occurs after the lysosomal fusion to ruffled border membrane (Supplementary Fig. 6).

### Coronin 1A inhibits LC3-I-to-II conversion and LC3 localization at ruffled border

**Figure 4:**
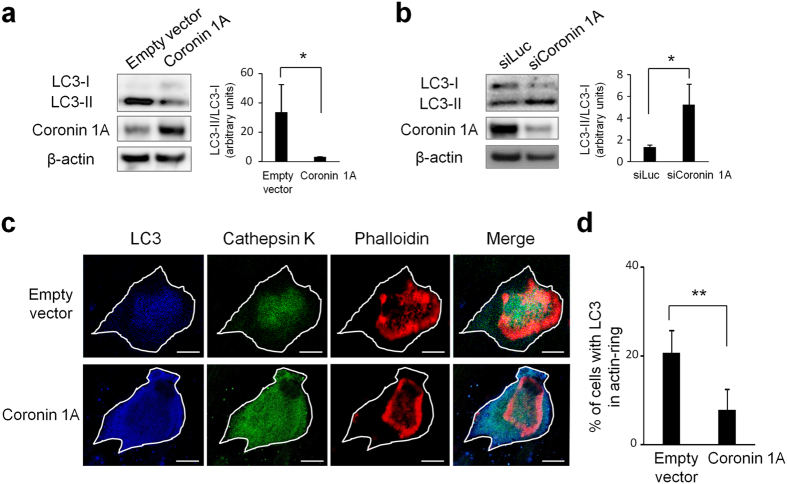
Coronin 1A regulated the lipidation and localization of LC3 in osteoclasts. (**a**) The amount of LC3 in cell lysates. Cell lysates of empty vector (control) or coronin 1A-overexpressing osteoclasts were analysed by immunoblotting. Right: The ratio of LC3-II to LC3-I was quantified from three independent experiments using ImageJ. Unprocessed original scans of blots are shown in Supplementary Fig. 11. *P < 0.05, (Student’s t-test). (**b**) Cell lysates of luciferase- or coronin 1A-knockdown osteoclasts were analysed by immunoblotting. Right: The ratio of LC3-II to LC3-I was quantified from three independent experiments using ImageJ. Unprocessed original scans of blots are shown in Supplementary Fig.11. *P < 0.05, (Student’s t-test). (**c**) Representative confocal images of control- and coronin 1A-overexpressing osteoclasts; actin, red; LC3, blue; cathepsin K, green; cell perimeters, white. Scale bars, 20 μm. (**d**) Percentage of control or coronin 1A-overexpressing osteoclasts with LC3 within the actin ring; data are presented as mean ± SD, n = 35 **P < 0.01, (Student’s t-test). Data are representative of three experiments.

Next, we attempted to clarify which mechanisms underlie the coronin 1A suppression of cathepsin K secretion. The previous study indicated that autophagic component LC3, one of eight mammalian Atg8 family of proteins, is converted from its cytosolic LC3-I form into LC3-II, which is a C-terminally phosphatidylethanolamine-conjugated form; and this post-translational modification process, called lipidation, is essential for both LC3 localization at ruffled border and lysosomal secretion from ruffled border^21^. Therefore, we quantified the production of LC3-II and examined LC3 localization in osteoclast-lineage cells using immunoblotting and confocal microscopy, respectively. LC3-II levels in osteoclasts were decreased by coronin 1A overexpression and were increased by the coronin 1A knockdown (Fig. 4a,b), suggesting the importance of coronin 1A for LC3 lipidation in osteoclasts. Moreover, enforced coronin 1A expression inhibited the localization of LC3 within actin ring (Fig. 4c,d). These results indicate that coronin 1A regulates LC3 lipidation and its localization at the osteoclast ruffled border.

As previously reported, small guanosin-5′-triphosphatase (GTPases) Rab7 and Rab27a are necessary for ruffled border formation and/or lysosomal secretion in osteoclasts^12^,^13^. Furthermore, the localization of Rab7 and LC3 are abnormal, and cathepsin K secretion is significantly decreased in Atg5-deficient osteoclasts^21^. Here, we asked whether coronin 1A overexpression could affect Rab7 or Rab27a in osteoclasts. The expression levels of Rab7 and Rab27a were not affected by coronin 1A overexpression (Supplementary Fig. 7a). Rab7 was observed principally at ruffled border in coronin 1A-overexpressing osteoclasts, and the co-localization with F-actin was evident both in coronin 1A-overexpressing and control osteoclasts (Supplementary Fig. 7b). In addition, Rab27a localization at cytoplasm was not also changed by coronin 1A overexpression (Supplementary Fig. 7b). These results indicated that coronin 1A specifically regulates LC3 lipidation and localization at ruffled border, but not Rab7 and Rab27a at least in osteoclasts.

### Coronin 1A regulates osteoclastic bone resorption activity via the actin-related mechanism(s)

**Figure 5:**
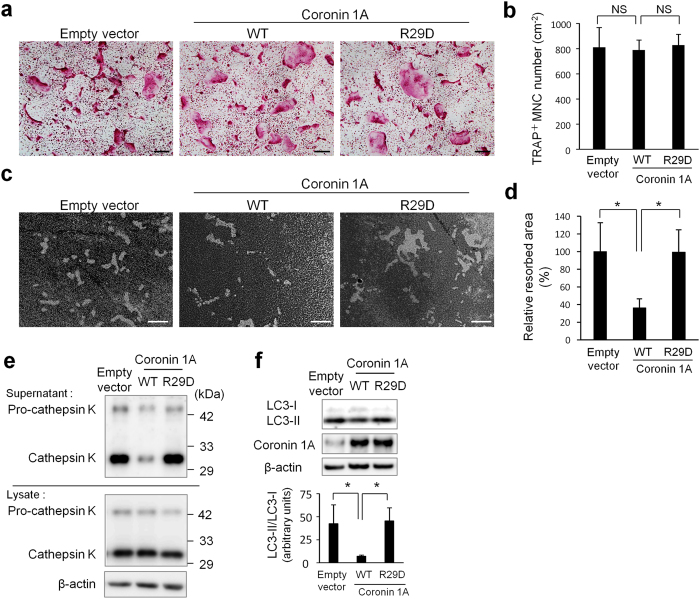
Coronin 1A regulated bone resorption via actin-dependent mechanism. (**a**–**f**) BMMs were transduced with empty vector (control) or coronin 1A (wild-type or R29D mutant)-expressing lentivirus, and then were cultured with M-CSF (10 ng/mL) and RANKL (50 ng/mL) to differentiate into osteoclasts. (**a**,**b**) Osteoclasts were fixed and stained for TRAP. The numbers of TRAP^+^ MNCs (>3 nuclei) were counted. Data are means ± SD of three independent experiments. Scale bars, 200 μm. (**c**,**d**) The osteoclasts were removed from the bone biomimetic synthetic surface plate and resorption pit areas were visualized by von Kossa staining and analysed by using the MetaMorph software. Data are means ± SD of three independent experiments. *P < 0.05 for the indicated comparisons. Scale bars, 50 μm. (**e**) The amount of cathepsin K in cell lysates and supernatants. Cell lysates and supernatants were analysed by immunoblotting. Unprocessed original scans of blots are shown in Supplementary Fig. 11. (**f**) The amount of LC3 in cell lysate. Right: The ratio of LC3-II to LC3-I was quantified from three independent experiments using ImageJ. Unprocessed original scans of blots are shown in Supplementary Fig. 11. *P < 0.05 for the indicated comparisons. Data are representative of three experiments.

Coronin 1A has been known to interact with cytoskeleton^24^,^30^. Therefore, we hypothesized that coronin 1A regulates LC3 lipidation and localization through actin-related modulation. We first constructed a mutant of coronin 1A (with an R29D mutation) that is devoid of actin-binding ability^44^,^45^ and then examined its effect on osteoclastogenesis and bone resorption. Coronin 1A overexpression by lentivirus (Supplementary Fig. 8), regardless of the R29D mutation, did not affect osteoclastogenesis (Fig. 5a,b). However, interestingly, the R29D mutation in coronin 1A reversed the inhibitory effect of coronin 1A overexpression on osteoclastic bone resorption activity (Fig. 5c,d). Of note, suppression of LC3 lipidation and cathepsin K secretion by coronin 1A overexpression were also abolished with the actin-binding deficient R29D mutant (Fig. 5e,f). These results strongly indicated that the interaction between coronin 1A and actin is crucial for coronin 1A-mediated suppression of bone resorption.

### Effect of enforced expression coronin 1A on lipopolysaccharide (LPS)-induced bone resorption *in vivo*

**Figure 6:**
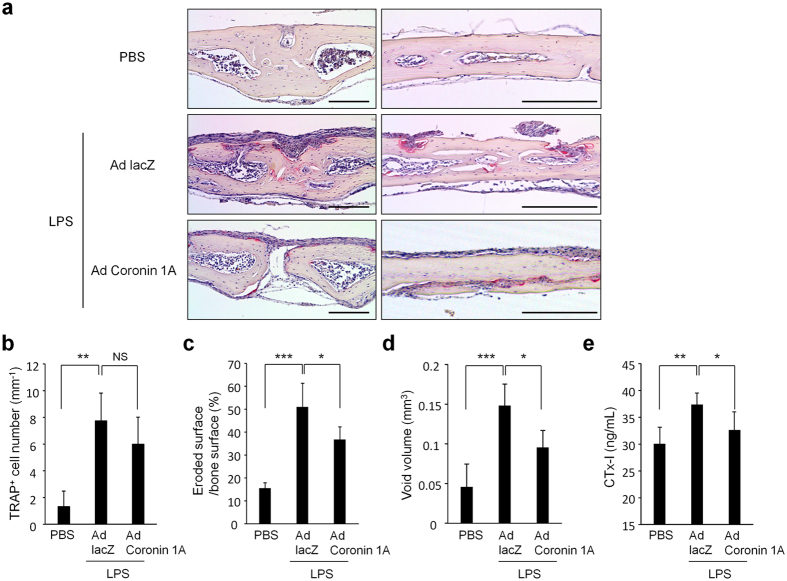
Enforced-coronin 1A suppressed LPS-induced bone resorption. Adenovirus expressing LacZ (Ad LacZ) or coronin 1A (Ad coronin 1A) and lipopolysaccharide (LPS) were injected onto calvariae in mice. (**a**–**c**) TRAP staining was performed on sections of calvarial bones. Scale bars, 200 μm. Data are means ± SD from four mice for each condition. (**d**) The void volume on the surface of the calvarial bone was determined by micro-computed tomography. Data are means ± SD, n = 4, PBS or Ad LacZ-injected mice, n = 5, Ad coronin 1A injected mice. (**e**) The concentration of serum CTx-I was measured using ELISA. Data are means ± SD from seven mice for each condition. (**b**–**e**) The overall difference between the groups was determined by one-way analysis of variance (ANOVA). Post hoc multiple comparisons were made using the Dunnett test. *P < 0.05, **P < 0.01, ***P < 0.005. Data are representative of three experiments.

Finally, we assessed whether enforced expression of coronin 1A would affect the severity of LPS-induced bone resorption in a mouse model. Mice were injected with LPS and adenovirus expressing LacZ (Ad LacZ: control) or coronin 1A (Ad coronin 1A), and we found that although the number of TRAP-positive cells on the bone surface was only slightly affected (Fig. 6a,b), Ad coronin 1A-injected mice showed less susceptibility to LPS-induced bone loss compared to Ad LacZ-injected mice; i.e., histological analysis (Fig. 6c), micro computed tomography (Fig. 6d) and the levels of serum CTx-I (collagen type 1 cross-linked C-telopeptide: a marker for bone resorption) (Fig. 6e) showed a significant decrease in bone resorption in the Ad coronin 1A-injected mice, suggesting that the coronin 1A-mediated machinery also operates *in vivo* and plays a crucial role in regulating bone resorption.

## Discussion

In the context of osteoclastic secretion of bone-degrading enzymes, the precise mechanism of the trafficking and fusion of lysosomal cargo to the ruffled border membranes remains poorly understood. In the present study, we revealed that coronin 1A is a negative regulator of cathepsin K secretion in osteoclast-lineage cells. Interestingly, coronin 1A prevented LC3 lipidation and LC3 localization to the ruffled border membranes, and the R29D mutation in coronin 1A reversed the inhibitory effects, indicating that coronin 1A attenuates the lipidation-mediated LC3 sorting through its binding capacity to actin.

Autophagy-related proteins have been shown to be responsible for lysosomal secretion in osteoclasts and melanocytes^21^,^46^,^47^. Actually, the autophagy-related proteins Atg5, Atg7, Atg4B and LC3, a member of the Atg8 family proteins, have been shown to be important in osteoclastic bone resorption, and LC3 lipidation and LC3 localization at ruffled border membrane were shown to be critical for efficient cathepsin K secretion in osteoclasts^21^. In addition, the deletion of coronin 1A was reported to enhance the recruitment of LC3 to phagosomes in macrophages^26^. Moreover, the small GTPase Rab7 has been identified as an essential component for both autophagosome fusion to lysosomes in variable cells and ruffled border formation in osteoclasts^12^,^48^,^49^.These reports collectively suggested that the autophagic pathway and the lysosomal secretion of bone-degrading enzymes in osteoclasts might share common molecular mechanisms, and it is likely that coronin 1A inhibits cathepsin K secretion by suppressing the cellular autophagic machinery. However, notably, knocking out the *Map1lc3b* gene (encoding LC3-β, a representative member of MAP1LC3/LC3 protein family in mammals^21^,^50^) did not affect osteoclast phenotype^21^, perhaps reflecting functional redundancy among Atg8 family members, such as GABARAP and GATE-16^21^,^50^,^51^. In accordance with these reports, osteoclastogenesis, cathepsin K-secretion and bone resorption activity of osteoclasts were not significantly changed by siRNA-mediated knockdown of LC3-β (Supplementary Fig. 9a–c). Future studies are needed to evaluate the significance of LC3 pathway and interplay among the Atg8 family proteins. During autophagosome formation, Atg12 is conjugated to Atg5 irreversibly, and the Atg12–Atg5 conjugate contributes to LC3 lipidation with its E3 ligase-like function^52^,^53^,^54^. Atg16L further forms a complex with the Atg12–Atg5 conjugate and facilitates LC3 lipidation^52^,^53^,^54^. Therefore, it may be possible that actin filament-bound coronin 1A attenuates the function of Atg12–Atg5 complex and/or Atg16L by yet unknown mechanisms.

Coronin 1A was also identified as an F-actin stabilizer against depolymerization^55^. Moreover, F-actin stabilization on phogosomal membrane by coronin 1A is required to block phagosome–lysosome fusion^25^,^56^. Therefore, it is possible that coronin 1A not only prevents LC3 lipidation and localization via the actin-related mechanism, but also protects plasma membranes (other than ruffled border membranes) from fusing with lysosomal vesicles through stabilizing actin cytoskeleton to prevent aberrant release of lysosomal enzymes. Further studies are required to prove this hypothesis.

It is noteworthy that previous studies have shown that coronin 1A plays a role in calcium mobilization as well as phagosome/endosome fusion to lysosomes^25^,^27^. Although calcium mobilization is a prerequisite for the calcium–calcineurin–NFATc1 signalling, the modulation of coronin 1A expression did not affect the NFATc1 activation and osteoclast differentiation. This fact suggests that coronin 1A-independent calcium–calcineurin signalling operates in osteoclastogenesis or that the existence of other coronin family proteins simply masks the gain- and loss-of-function phenotype(s) of coronin 1A mutant cells in osteoclastogenesis. In fact, there are seven coronin genes in mammals. Although coronin family members share a common structure, i.e. WD repeats and a carboxy-terminal coiled-coil domain, functional redundancy among coronin family proteins has not been fully characterized. Indeed, although five coronin members, coronin 1A, 1B, 1C, 2A and 7, are expressed in macrophages, only coronin 1A has been identified as a regulator of phagosomal membrane fusion with lysosomes^25^,^57^. This may explain the strong phenotype in coronin 1A mutant cells concerning osteoclast function but not osteoclastogenesis. To completely elucidate the functional redundancy among coronin family proteins in osteoclast differentiation and function, further studies will be required.

We showed that coronin 1A knockdown or overexpression strongly affected bone resorption. Nevertheless, to date, an abnormal bone phenotype in coronin 1A-deficient mice has never been reported. The previous study showed that autophagic protein Atg5-deficient osteoclasts exhibit a remarkable reduction in osteoclastic bone resorption activity *in vitro*; but Atg5-deficient mice only showed a minimal increase in bone mass^21^. However, Atg5-deficient mice were protected from ovariectomy- or ageing-induced bone loss^21^, suggesting that a defect in autophagic machinery may cause a strong defect in osteoclasts *in vitro*; however, for unknown reasons, bone remodeling appears normal *in vivo* unless osteoclasts are activated by a relevant ‘challenge’. Accordingly, bone mass in coronin 1A-deficient mice also might be affected by pathological challenges.

In the present study, we showed that RANKL stimulation downregulated coronin 1A expression (Fig. 1). The coronin 1A promoter region contains a binding site for the Maf family of transcription factors, as detected by using the MATCH program and the TRANScription FACtor (TRANSFAC) database. MafB, one of the Maf family of transcription factors, is expressed in monocytes and macrophages and has been reported to negatively regulates RANKL-induced osteoclastogenesis^58^; the expression of MafB itself is downregulated by RANKL stimulation^59^. These data may imply the possible relationship between RANKL-induced suppression of MafB expression and the downregulation of coronin 1A during osteoclastogenesis.

Regarding the role of coronin 1A in osteoclast precursor cells, i.e., in monocyte/macrophage-lineage cells, it has been shown that the inflammatory stimulation-induced coronin 1A phosphorylation triggers cellular switching from phagocytosis to micropinocytosis, which enables efficient uptake and degradation of large amounts of infectious materials^60^,^61^, suggesting that coronin 1A is important for host defense against infection. How about the role of coronin 1A in premature osteoclasts then? Premature osteoclasts actually express a large quantity of cathpsin K in cells, however, they would not considerably secrete it until they reach a later stage of maturation and firmly stick to the bones. Given the high expression of coronin 1A in macrophages and premature osteoclasts, it raises the intriguing possibility that coronin 1A operates mainly in those cells to tightly regulate lysosomal secretion of vital enzymes such as cathepsins. However, further studies are required to address this point.

In conclusion, we have demonstrated that coronin 1A and its binding to actin play an important role, possibly in concert with autophagic machinery, in regulating cathepsin K secretion in osteoclast-lineage cells. The present study thus provides a novel therapeutic target for bone diseases such as osteoporosis and rheumatoid arthritis.

## Materials and Methods

### Mice

C57BL/6 J mice were purchased from CLEA Japan (Japan). All experimental protocols were approved by the Animal Research Committee in Kyoto University, and this study was carried out in accordance with the Regulation on Animal Experimentation in Kyoto University.

### *In vitro* osteoclastogenesis

Bone marrow cells derived from mice were seeded (2.5 × 10^5^ cells per well in a 24 well plate) and cultured in α-minimum essential medium (α-MEM) with 10% fetal bovine serum (FBS) containing 10 ng/mL recombinant M-CSF (PeproTech). After two days, adherent cells were used as BMMs. These osteoclast progenitor cells were further cultured in the presence of 50 ng/mL recombinant RANKL (PeproTech) and 10 ng/mL recombinant M-CSF to generate mature osteoclasts for 48–72 h. The presence of osteoclasts was confirmed by TRAP staining. TRAP-positive multinuclear cells (more than three nuclei) were counted.

### RNA interference

The stealth small interfering RNAs (siRNAs) targeting mouse coronin 1A and LC3-β were designed and synthesized by Invitrogen. The siRNA sequences are as follows: si-coronin 1A: 5′-UUGUCUACUCGUCCAGUCUUGCCUA-3′, si-coronin 1A: 5′-UGUAGAUUGUGUCCGGGUGCACAUC-3′ and si-LC3-β: 5′-CAGCUCAAUGCUAACCAAGCCUUCU-3′, si-luciferase (negative control): 5′-ACAUCACGUACGCGGAAUACUUCGA-3′. BMMs were transfected with siRNAs (40 nmol) with Lipofectamine RNAiMAX reagent (Invitrogen), according to the manufacturer’s protocol. After 48 h, the BMMs were cultured with M-CSF and RANKL for osteoclast generation or protein analysis.

### Lentiviral-mediated gene transfer

The recombinant lentivirus vector that carried either wild-type mouse coronin 1A (Coronin 1AA [wt]) or actin-binding defective mouse coronin 1A (Arg 29 to Asp mutant, Coronin 1A [R29D]) gene was constructed. To generate lentiviruses, 293 T cells were transfected with the lentivirus vector (CSII-CMV-MCS-IRES2-Venus), in combination with the packaging plasmid (pCAG-HIVgp) and the VSV-G- and Rev-expressing plasmid (pCMV-VSV-G-RSV-Rev) by calcium phosphate coprecipitation and incubated for 12 h. After removing the medium, cells were cultured in Dulbecco’s Modified Eagle Medium (DMEM) containing 10 μM forskolin for 48 h, and virus supernatants were collected. BMMs were transduced with virus supernatant for 24 h in the presence of 10 μg/mL polybrene (Sigma). The BMMs were cultured with M-CSF and RANKL for osteoclast generation.

### Real-time PCR

Total RNAs from the cultured cells were extracted with the RNeasy kit (Qiagen). First-strand cDNA was then produced from total RNA using Reverse Transcriptase SuperScript III (Invitrogen). *Coro1a, Nfatc1, Dcstamp, Atp6v0d2, Acp5* and *Ctsk* transcripts were quantified on a LightCycler 480 (Roche) using SYBR green. These transcripts were normalized to glyceraldehyde-3-phosphate dehydrogenase (*Gapdh*) transcripts. Primers for mouse *Coro1a* (forward 5′-TTCCCTCAAGGATGGCTACGT-3′ and reverse 5′-CTCCGGCCTAGCCATGTGAT-3′), mouse *Nfatcl* (forward 5′-GGTAACTCTGTCTTTCTAACCTTAAGCTC-3′ and reverse 5′-GTGATGACCCCAGCATGCACCAGTCACAG-3′), mouse *Ctsk* (forward 5′-GCCAGGATGAAAGTTGTATG-3′ and reverse 5′-CAGGCGTTGTTCTTATTCC-3′), mouse *Dcstamp* (forward 5′-TGTATCGGCTCATCTCCTCCAT-3′ and reverse 5′-GACTCCTTGGGTTCCTTGCTT-3′), mouse *Atp6v0d2* (forward 5′-GAAGCTGTCAACATTGCAGA-3′ and reverse 5′-TCACCGTGATCCTTGCAGAAT-3′), mouse *Acp5* (forward 5′-ACTTCCCCAGCCCTTACTACCG-3′ and reverse 5′-TCAGCACATAGCCCACACCG-3′), mouse *Gapdh* (forward 5′-TCACCATCTTCCAGGAGCGA-3′ and reverse 5′-GCATTGCTGACAATCTTGAGTGAG-3′).

### Immunoblot analysis

The cells were lysed with CelLytic M solution (Sigma-Aldrich). After centrifugation, the supernatant was collected, mixed with SDS sample buffer, and boiled for 5 min at 98 °C. The sample was applied to SuperSep Ace gel (Wako), separated by a standard SDS-PAGE method, and then transferred onto polyvinylidene fluoride membranes, blocked with 5% skim milk and incubated at 4 °C overnight with anti-Akt, anti-p-Akt (S473) anti-p38, anti-p-p38 (T180/Y182), anti-ERK, anti-p-ERK (T202/Y204), anti-JNK, anti-p-JNK (T183/Y185) (dilution 1:3,000; Cell Signaling Technology), anti-cathepsin K (dilution 1:1,000; Santa Cruz), anti-coronin1A, anti-rab7 (dilution 1:3,000; Abcam), anti-LC3 (HRP conjugate) (dilution 1:2,000; MBL), anti-rab27a (dilution 1:3,000; R&D) and anti-β-actin (HRP conjugate) (dilution 1:2,000; Cell Signaling Technology). The membranes were washed and incubated with horseradish peroxidase-conjugated secondary antibodies. Chemiluminescence was detected using ImmunoStar LD (Wako), and images were captured by using LAS-4000 mini (Fujifilm) or Amersham Imager 600 (GE Healthcare).

### Assesment of osteoclastic bone resorption activity

BMMs were cultured on the bone biomimetic synthetic surface plate and differentiation to osteoclasts was induced. The cells were removed from the plates, and bone pits generated by osteoclasts were stained and visualized by von Kossa staining. After washing the wells, pits were imaged by microscopy (Evos; Thermo Fisher Scientific). The pits were analysed using MetaMorph software.

### Immunofluorescence and microscopy

BMMs were cultured on glass or dentine slides and induced to differentiate into osteoclasts. Osteoclasts were fixed with 4% paraformaldehyde for 15 min then washed three times with phosphate buffered saline with Tween 20 (PBST). Cells were permeabilized in 0.1% Triton-X for 10 min and blocked for 1 h in PBST containing 2.5% bovine serum albumin (BSA) and 10% normal goat serum. Primary antibody diluted in Can Get Signal immunostain solution A (Toyobo) was added for 12 h at 4 °C, followed by 1 h incubation with secondary antibody together with phalloidin-647 (Abcam) and DAPI. The following antibodies were used: anti-coronin 1A (Abcam), anti-cathepsin K (Abcam), anti-LAMP1 (Cell Signaling Technology, Biolegend), anti-LC3 (MBL), anti-rab7 (Abcam), anti-rab27a (R&D), anti-mouse Alexa Fluor 488 and anti-rabbit Alexa Fluor 594 (both from Molecular Probes). Slides were imaged on LSM 710 (Zeiss) and analysed using MetaMorph software.

### Acridine orange staining assay

The acidification activity was measured with Acridine orange staining assay according to previously described methods^40^. Osteoclasts were incubated in α-MEM with 10% FBS containing 5 μg/mL Acridine orange (Dojindo) for 15 min at 37 °C, then washed three times with PBS. The cells were incubated in fresh medium without Acridine orange for 10 min at 37 °C and observed by confocal microscope (LSM 710 [Zeiss]) with an appropriate filter set (excitation: 450- to 490-nm band-pass filter and emission: 515- to 565-nm band-pass filter or 600-nm long-pass filter). Images were analysed by using MetaMorph software.

### LPS-induced bone resorption model

The recombinant adenovirus vector (pAdenoX-ZsGreen1) that carried mouse coronin 1A was constructed. To generate recombinant adenoviruses, Adeno-X 293 cells were transfected with the PacI-digested adenovirus by using Adeno-X™ Adenoviral System 3 (Clontech Laboratories) according to the manufacturer’s instructions. In total, 1 × 10^7^ infection forming units (IFU) of virus diluted in 50 μL of PBS were injected onto the calvariae four times at one day intervals. Mice were administered with a local calvarial injection of LPS at 1.5 mg/kg body weight three times at two days intervals. Seven days after the first injection, the calvariae were collected and fixed in 4% paraformaldehyde and analysed histologically. The number of TRAP-positive cells per millimetre of calvarial bone surface was counted. The eroded surface per bone surface (ES/BS, %) was analysed by using MetaMorph software.

### Micro-computed tomography analysis

Calvarial bones of the mice injected with adenovirus expressing LacZ or coronin 1A were analysed using ScanXmate-A100S Scanner (ComScanTechno, Yokohama, Japan) as described previously^62^. Three-dimensional microstructure images were reconstituted and the void volume on the surface of the calvarial bones was calculated by TRI/3D-BON software (RATOC System Engineering, Tokyo, Japan).

### Statistical analysis

Data were presented as means ± standard error of the mean (SEM) and compared using the Student’s t-test or one-way analysis of variance (ANOVA) plus post hoc multiple comparisons using the Dunnett test. A value of P < 0.05 was considered significant.

## Additional Information

**How to cite this article**: Ohmae, S. *et al*. Actin-binding protein coronin 1A controls osteoclastic bone resorption by regulating lysosomal secretion of cathepsin K. *Sci. Rep.* **7**, 41710; doi: 10.1038/srep41710 (2017).

**Publisher's note:** Springer Nature remains neutral with regard to jurisdictional claims in published maps and institutional affiliations.

## Supplementary Material

Supplementary Information
