# Supplementary material for: Actin-binding protein coronin 1A controls osteoclastic bone resorption by regulating lysosomal secretion of cathepsin K
Source: Sci Rep. 2017 Mar 16;7:41710. doi: 10.1038/srep41710 (PMC5353622; doi:10.1038/srep41710)
Supplement: Supplementary Information [file srep41710-s1.pdf]

Supplementary Information for

Actin-binding protein coronin 1A controls osteoclastic bone resorption by regulating lysosomal secretion of cathepsin K

Saori Ohmae, Naruto Noma, Masayasu Toyomoto,
Masahiro Shinohara, Masatoshi Takeiri, Hiroaki Fuji, Kenji Takemoto,
Keiko Iwaisako, Tomoko Fujita, Norihiko Takeda, Makoto Kawatani,
Mineyoshi Aoyama, Masatoshi Hagiwara,
Yasushi Ishihama, Masataka Asagiri

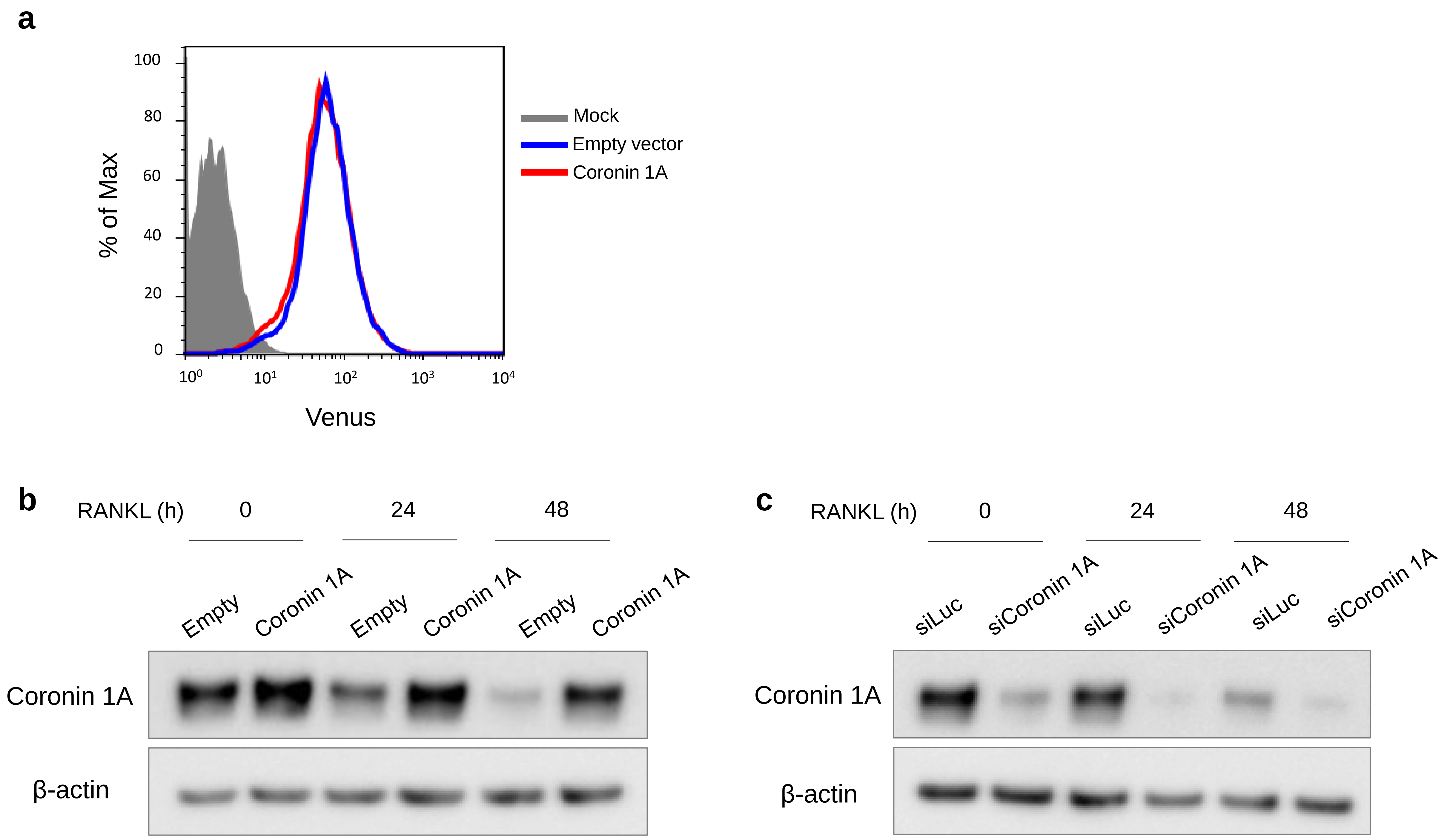

Supplementary Figure 1. Confirmation of coronin 1A overexpression or its knockdown
(**a, b**) BMMs were transduced with CSII-CMV-MCS-IRES2-Venus (empty vector) lentivirus or with lentivirus expressing coronin 1A. (**c**) BMMs were transfected with luciferase- or coronin 1A-specific siRNA. (**a**) Percentage of the Venus$^+$ (infected) BMMs using FACS analysis. (**b, c**) BMMs were cultured with M-CSF (10 ng/mL) and RANKL (50 ng/mL) to differentiate into osteoclasts and the expression of coronin 1A at protein level were analysed by immunoblot. Data are representative of three experiments. Unprocessed original scans of blots are shown in Supplementary Fig. 12.

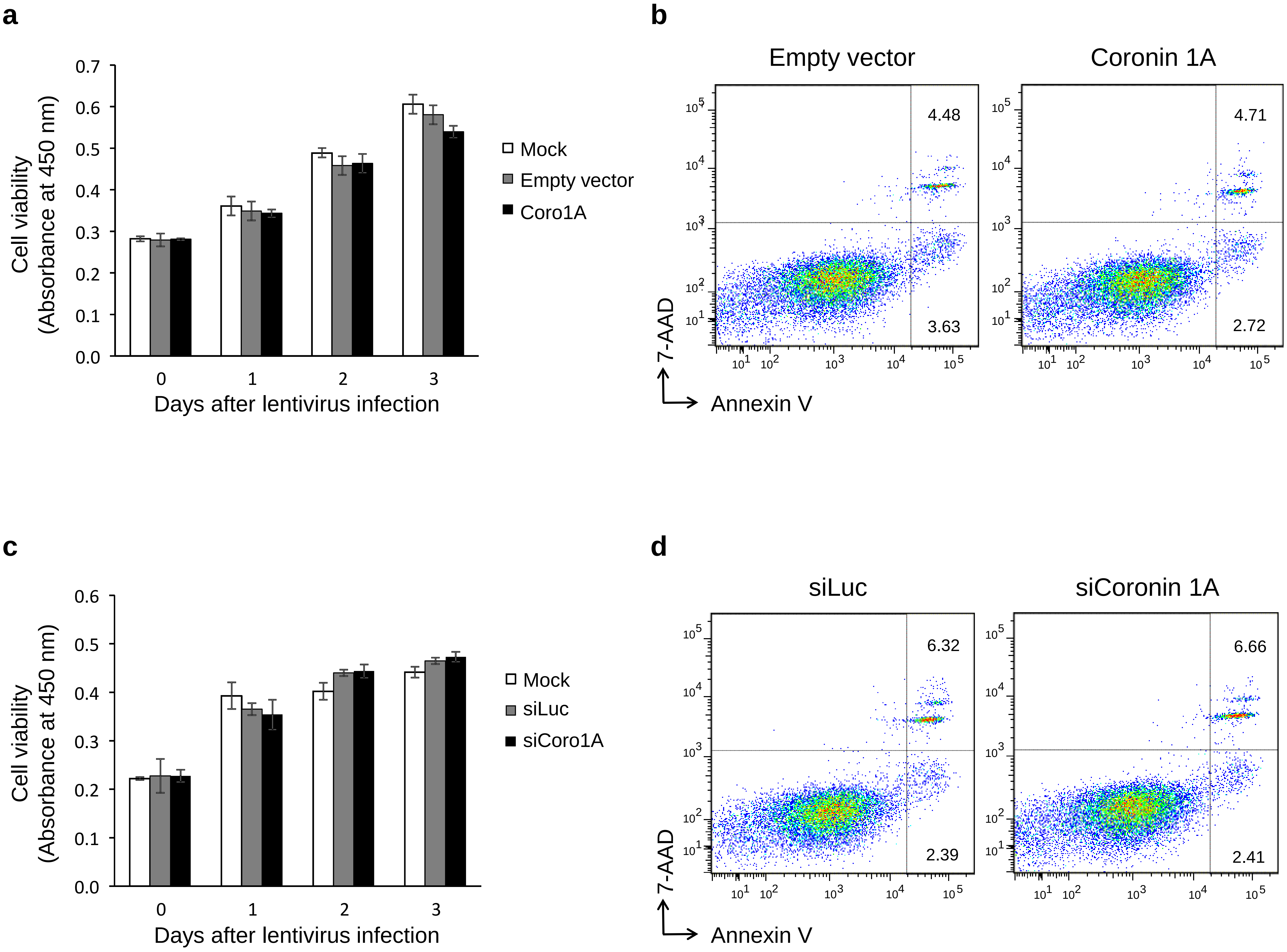

Supplementary Figure 2. Effects of coronin 1A overexpression or knockdown on cell proliferation and survival in bone marrow macrophages (BMMs)

(**a, b**) BMMs were transduced with CSII-CMV-MCS-IRES2-Venus (empty vector) lentivirus or with lentivirus expressing coronin 1A. (**c, d**) BMMs were transfected with luciferase- or coronin 1A-specific siRNA. (**a, c**) No difference in cell proliferation was observed by overexpression or knockdown of coronin 1A. The BMMs were incubated with 2-(2-methoxy-4-nitrophenyl)-3-(4-nitrophenyl)-5-(2,4-disulfophenyl)-2H-tetrazolium (WST-8) reagent in culture medium. Cell viability was determined using absorbance readings at 450 nm. (**b, d**) No significant difference on apoptosis rate was observed by overexpression or knockdown of coronin 1A. The cells were stained with AnnexinV and 7-AAD, and then late-stage apoptotic and necrotic cells (AnnexinV^{+} 7-AAD^{+}) and the early-stage apoptotic cells (AnnexinV^{+} 7-AAD^{-}) were evaluated. Representative FACS data and the percentage of the AnnexinV^{+} 7-AAD^{+} cells and AnnexinV^{+} 7-AAD^{-} cells are shown. Data are representative of three experiments.

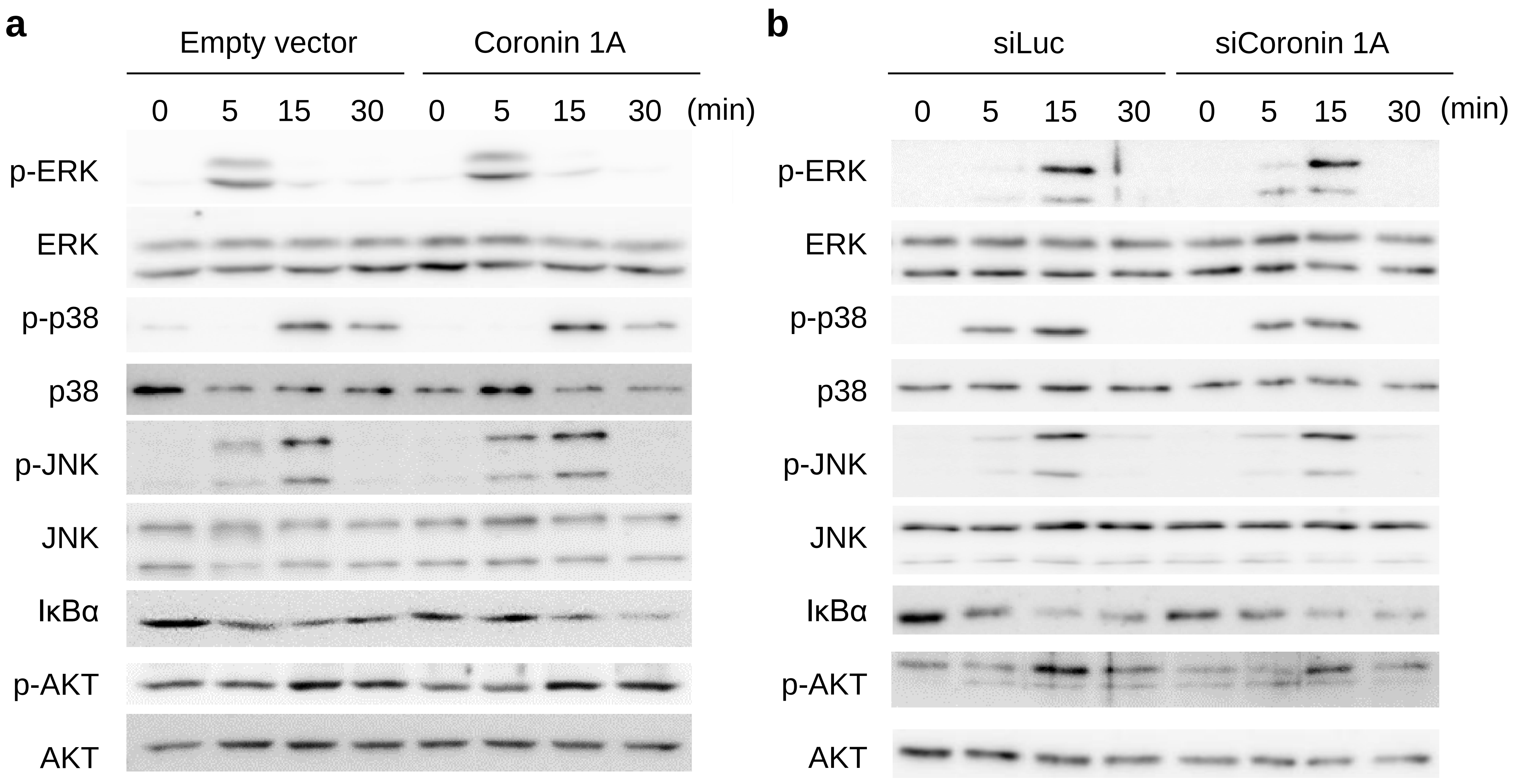

Supplementary Figure 3. Effect of coronin 1A overexpression or knockdown on RANKL-dependent NF-κB, MAPK and AKT signalling activation

(**a**) BMMs were transduced with empty vector- or coronin 1A-expressing lentivirus and stimulated for 0, 5, 15, or 30 min with RANKL. We compared the phosphorylation of ERK, p38, JNK, AKT and the degradation of IκBα between empty vector- and coronin 1A-overexpressing BMMs. (**b**) BMMs were transfected with luciferase- or coronin 1A-siRNA and were stimulated with RANKL. Luciferase-specific siRNA was used as negative control. Immunoblots in all panels are representative of three independent experiments. Unprocessed original scans of blots are shown in Supplementary Fig. 13.

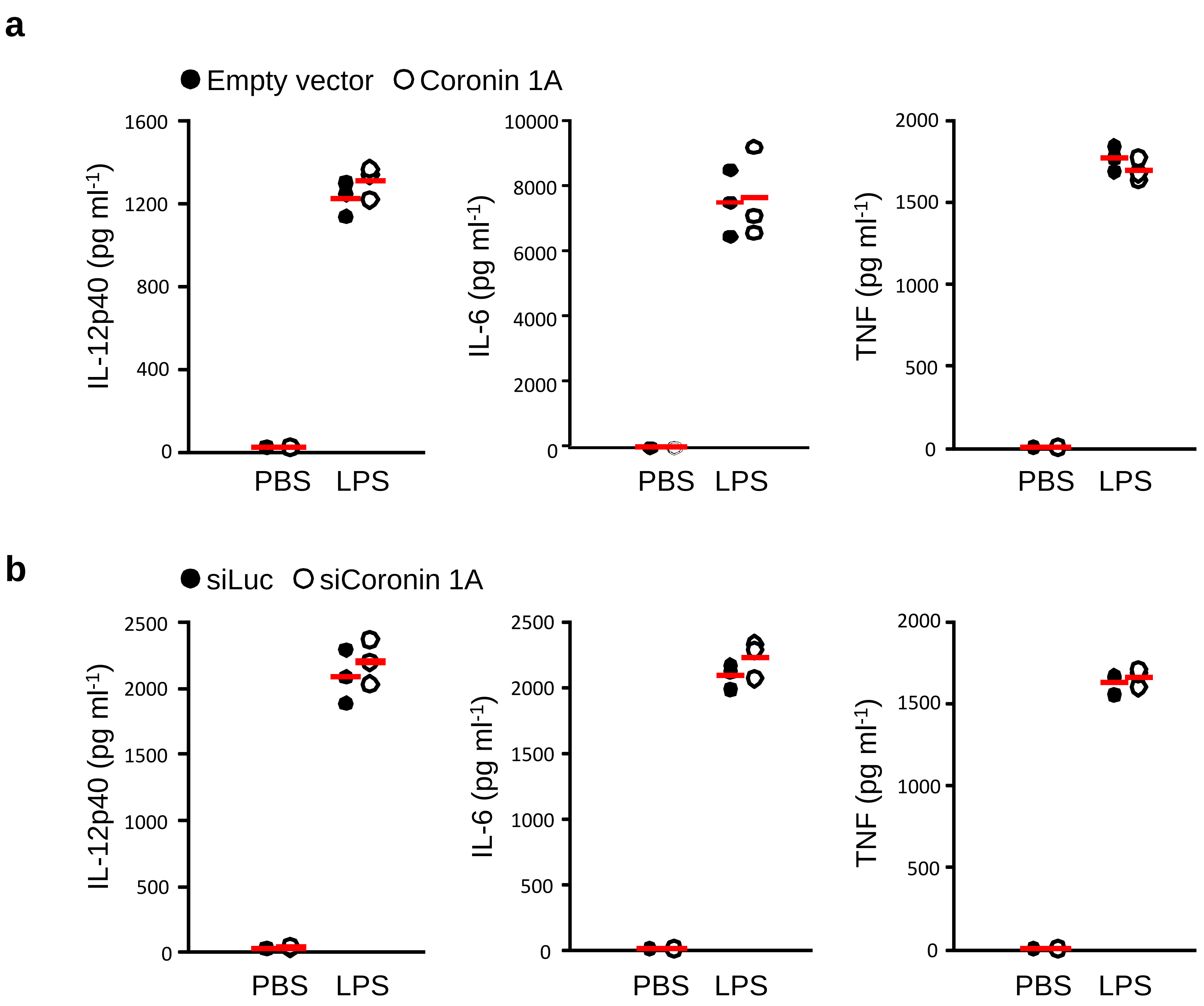

Supplementary Figure 4. Effect of coronin 1A overexpression or knockdown on cytokine production
(**a**) ELISA of cytokines secreted by coronin 1A-overexpressing BMMs. BMMs which were transduced with empty vector- (black circles) or coronin 1A (white circles)-expressing lentivirus were stimulated with 500 ng/mL lipopolysaccharide (LPS) for 24 h. The concentrations of IL-12/IL-23p40, IL-6 and TNF in culture supernatants were measured using ELISA. (**b**) ELISA of cytokines secreted by BMMs transfected with luciferase- (black circles) or coronin 1A- (white circles) specific siRNA were stimulated with 500 ng/mL LPS for 24 h, and the concentrations of cytokines in culture supernatants were measured using ELISA. Data are representative of three experiments.

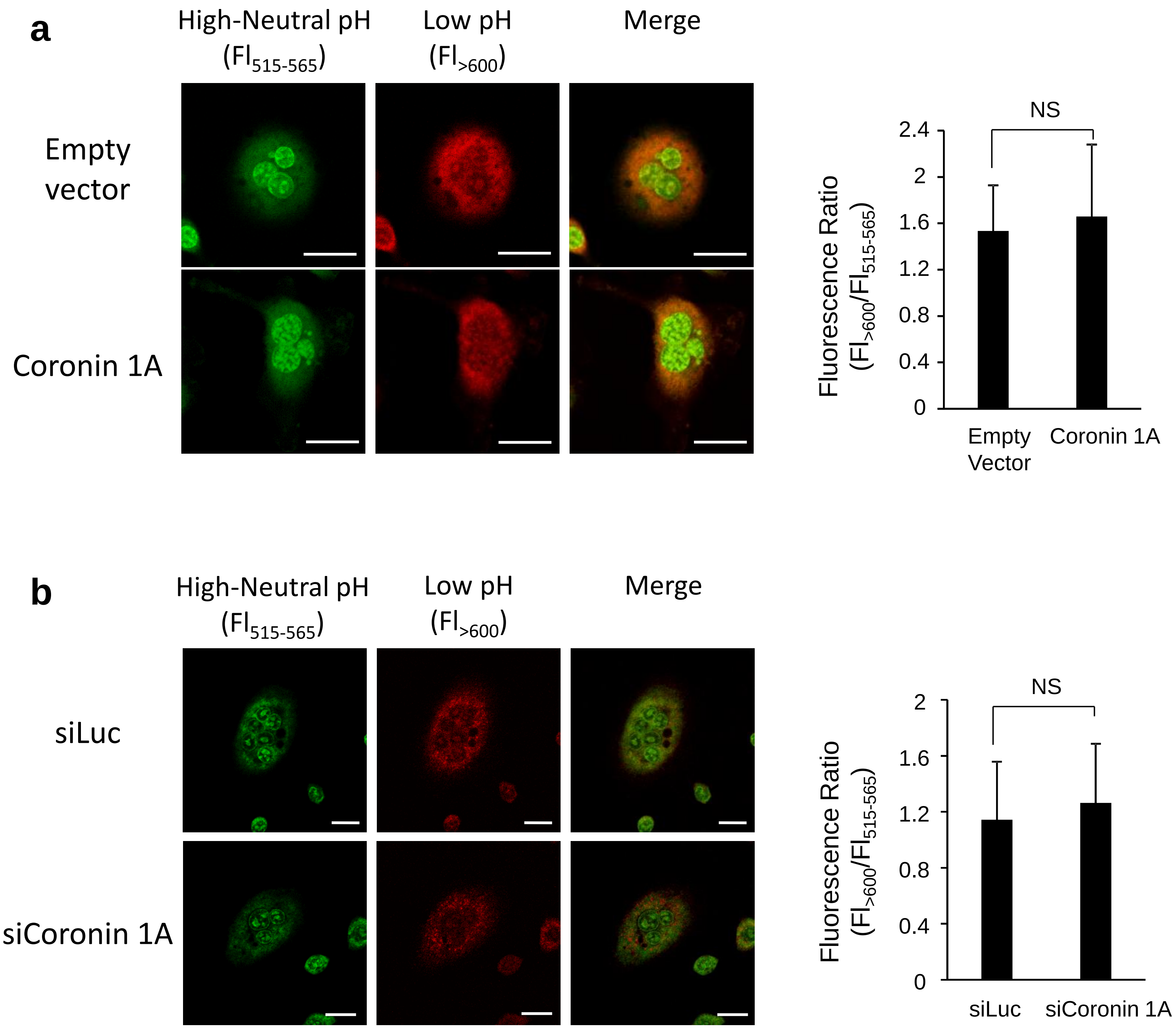

Supplementary Figure 5. Effects of coronin 1A overexpression or knockdown on extracellular acidification in osteoclasts

(**a**) BMMs were transduced with CSII-CMV-MCS-IRES2-Venus (empty vector) lentivirus or with lentivirus expressing coronin 1A. (**b**) BMMs were transfected with luciferase- or coronin 1A-specific siRNA. (**a, b**) No difference on extracellular acidification was observed by overexpression or knockdown of coronin 1A. The BMMs were cultured with M-CSF (10 ng/mL) and RANKL (50 ng/mL) to induce osteoclastogenesis. The osteoclasts were stained with acridin orange (5 μg/mL) and observed by confocal microscopy with an appropriate filter set (excitation: 450- to 490-nm band-pass filter and emission: 515- to 565-nm band-pass filter or 600-nm long-pass filter). Scale bars, 20 μm. The ratio of red fluorescence >600 nm intensity (acidic condition; low pH) to green fluorescence at 515- to 565-nm intensity (high to neutral pH) was calculated to estimate extracellular acidification by using MetaMorph software. Data are presented as mean ± SD, n = 25 in each group. (Student's t-test), NS: not-statistically significant. Data are representative of three experiments.

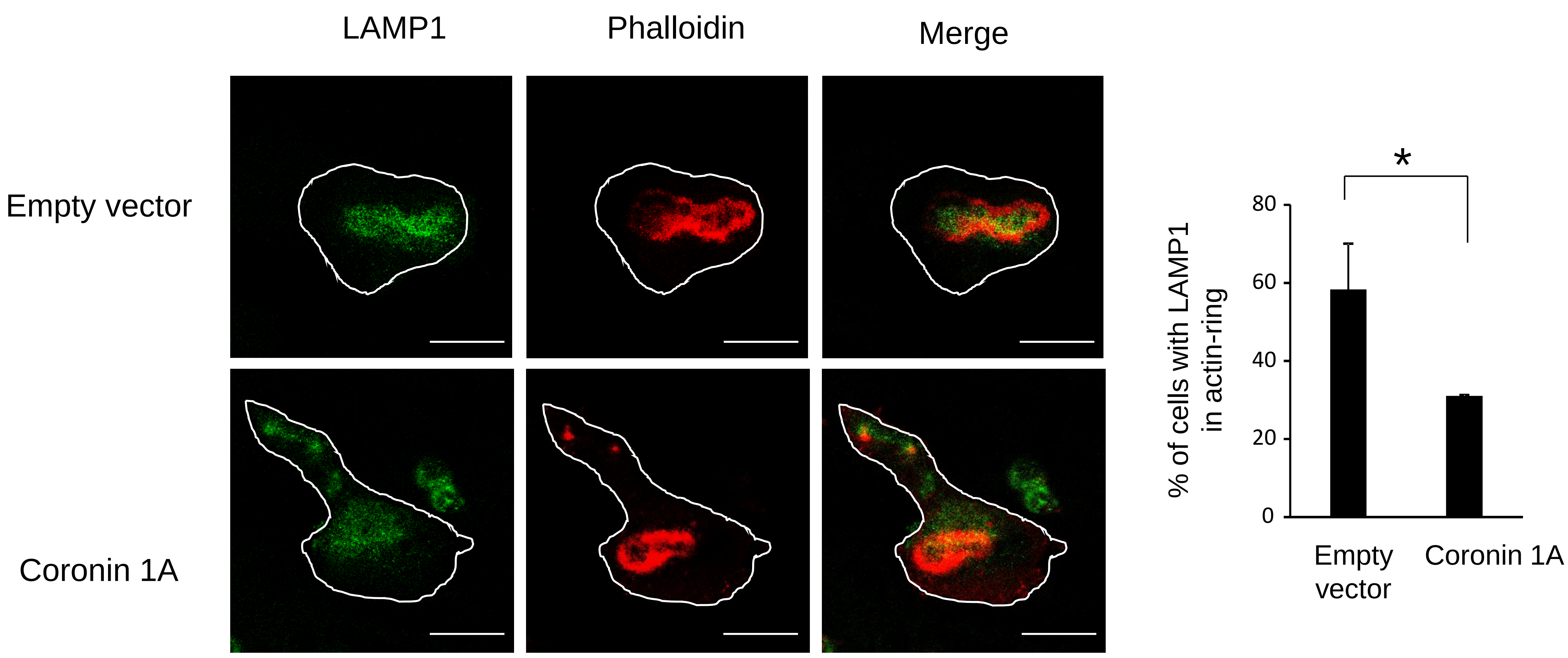

Supplementary Figure 6. The localization of LAMP1 in coronin 1A-overexpressing osteoclasts
Representative confocal images of empty vector (control)- or coronin 1A-overexpressing osteoclasts; actin, red; LAMP1, green; cell perimeters, white. Scale bars, 20 μm. Percentage of cells with lysosomal-associated membrane protein 1 (LAMP1) localized in actin-ring of control- or coronin 1A-overexpressing osteoclasts; data are presented as mean ± SD, n = 25 in each group. *P < 0.05, (Student's t-test). Data are representative of three experiments.

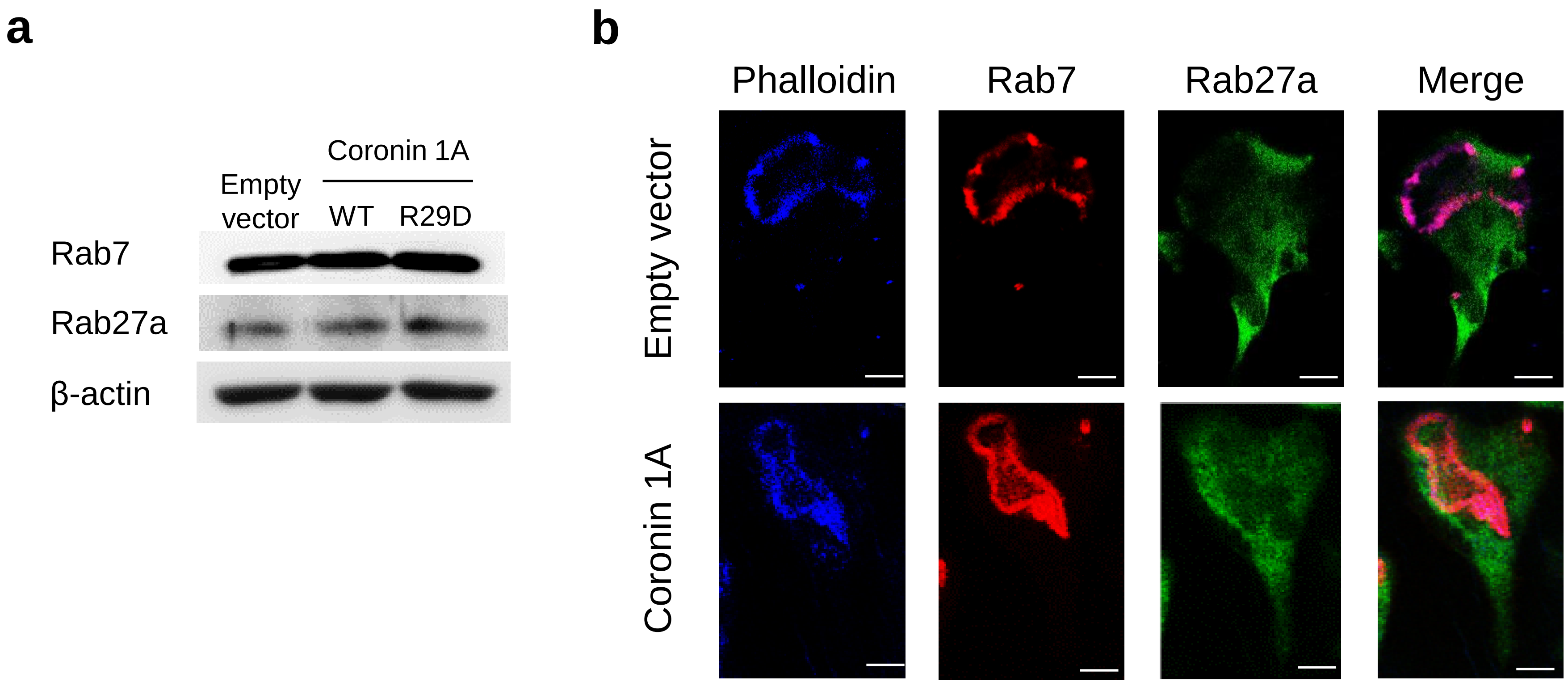

Supplementary Figure7. Expression and localization of Rab7 and Rab27a
(**a**) The expression of Rab7 and Rab27a in an empty vector- (control) or coronin 1A-overexpressing osteoclasts. BMMs were transduced with empty vector- or coronin 1A-expressing lentivirus, and then were differentiated to osteoclasts in the presence of M-CSF and RANKL. The expression of Rab7 and Rab27a were analyzed by immunoblot. Unprocessed original scans of blots are shown in Supplementary Fig. 14. (**b**) The localization of Rab7 and Rab27a in empty vector- or coronin 1A-overexpressing osteoclasts. The osteoclasts were fixed and stained with phalloidin, anti-Rab7 or anti-Rab27a, and their localization was observed by confocal microscopy. Scale bars, 20 μm. Data are representative of three experiments.

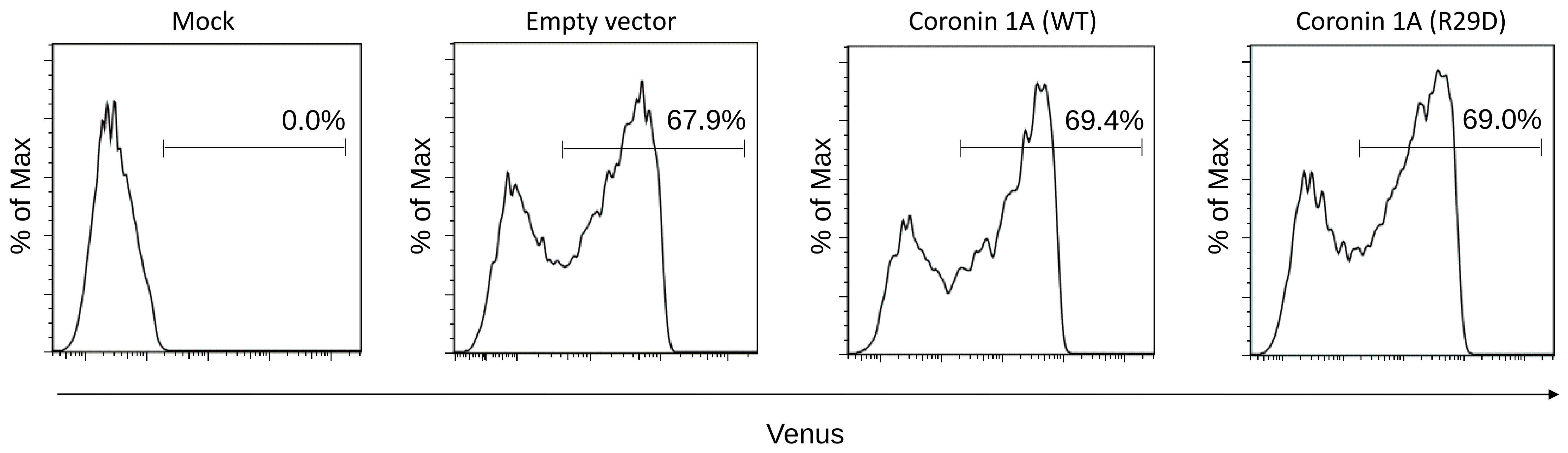

Supplementary Figure 8. Infection efficiency of bone marrow macrophages (BMMs) by lentiviral vectors
BMMs were transduced with CSII-CMV-MCS-IRES2-Venus (empty vector) lentivirus, or with lentivirus expressing coronin 1A (wild type) or actin binding-deficient mutant of coronin 1A (R29D). Representative fluorescence-activated cell sorting (FACS) data are shown; the numbers in the histograms indicate percentage of the Venus^{+} (infected) BMMs using FACS analysis. Data are representative of three experiments.

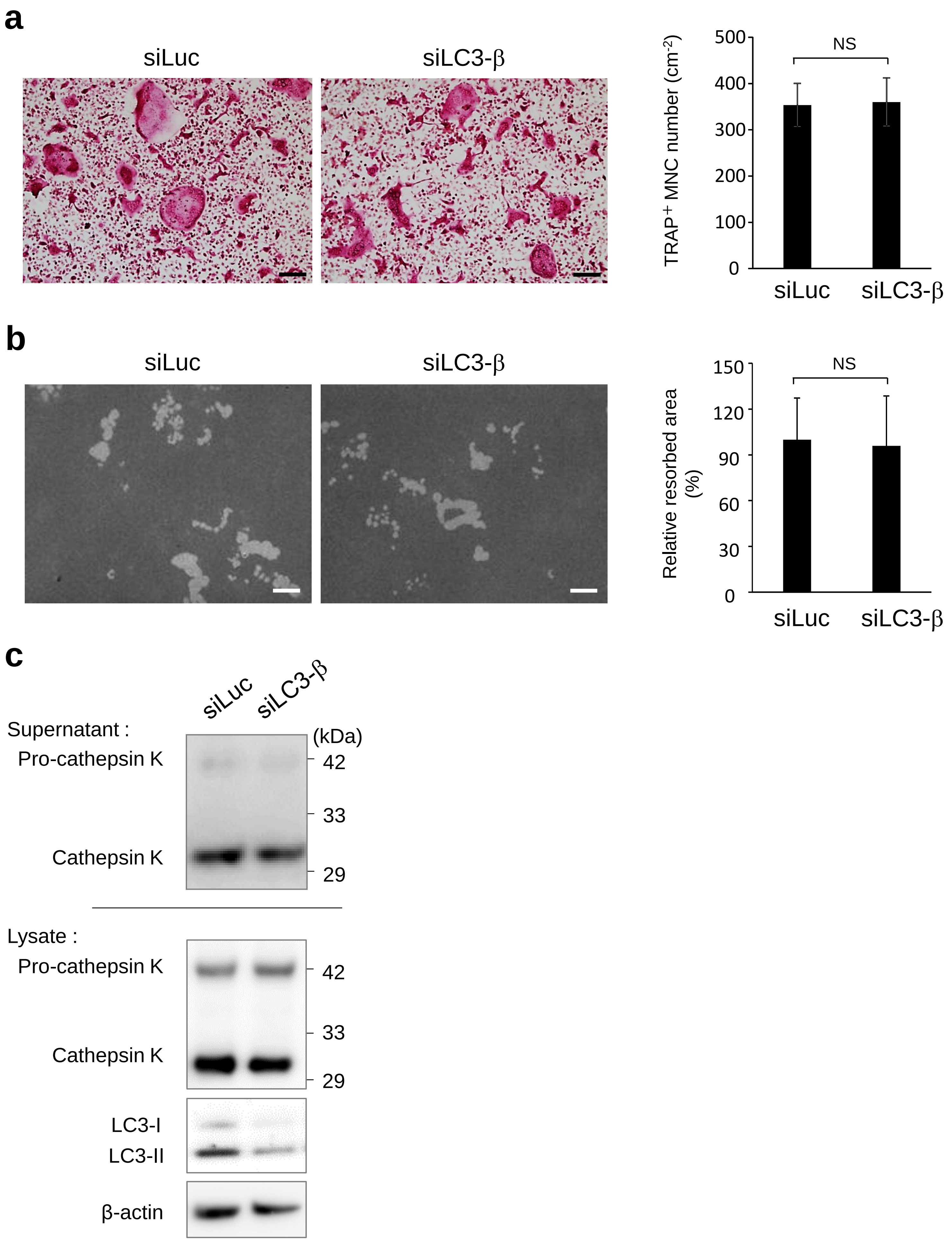

Supplementary Figure 9. Effects of LC3-β knockdown on osteoclast differentiation and bone resorption
(**a-c**) BMMs were transfected with luciferase- or LC3-β-specific siRNA. (**a**) The numbers of TRAP-positive MNCs (>3 nuclei) were counted. Scale bars, 200 μm. Data were means ± SD of three independent experiments. (Student's t-test), NS: not-statistically significant. (**b**) The bone resorption areas were visualized by von Kossa staining and analysed by using the MetaMorph software. Scale bars, 50 μm. Data were means ± SD of three independent experiments. (Student's t-test), NS: not-statistically significant. (**c**) The amount of cathepsin K in cell lysates and supernatants were analysed by immunoblot. Data are representative of three experiments. Unprocessed original scans of blots are shown in Supplementary Fig. 14.

Fig. 1b

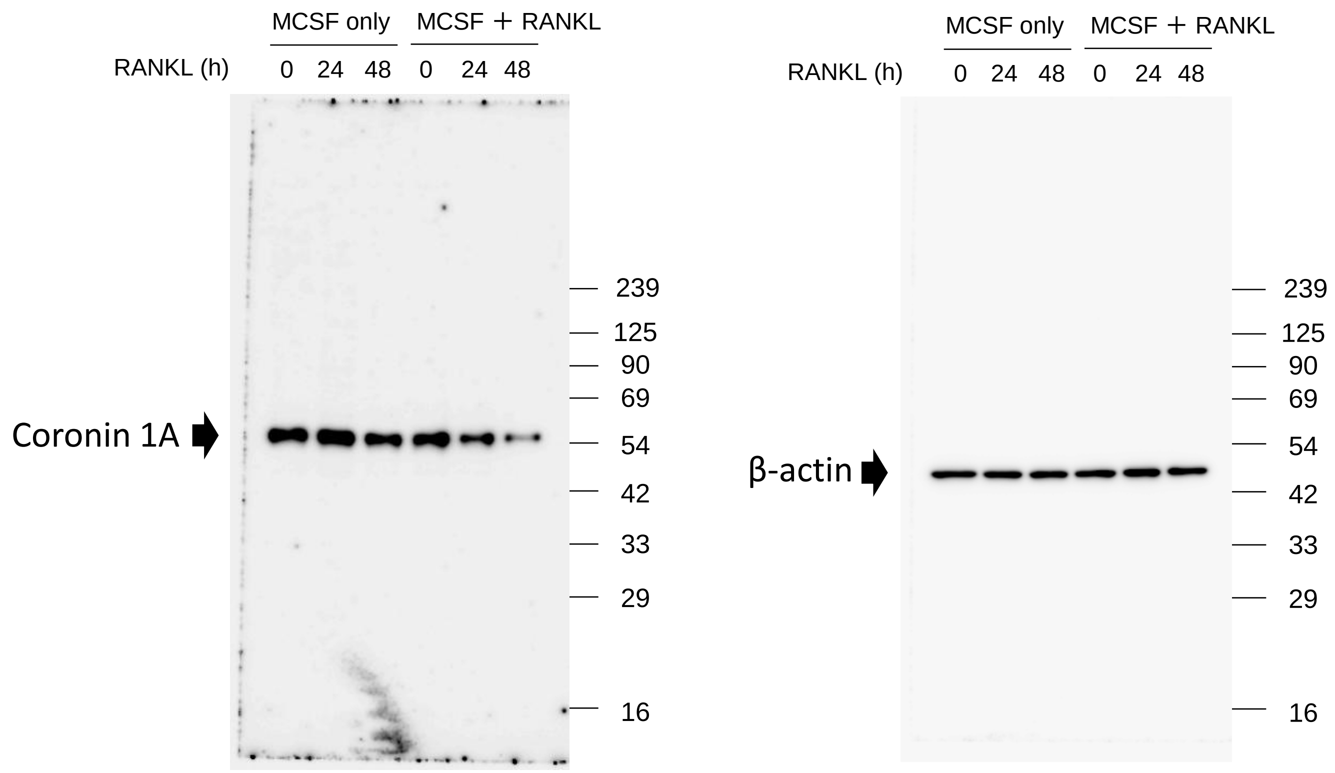

Fig. 3c

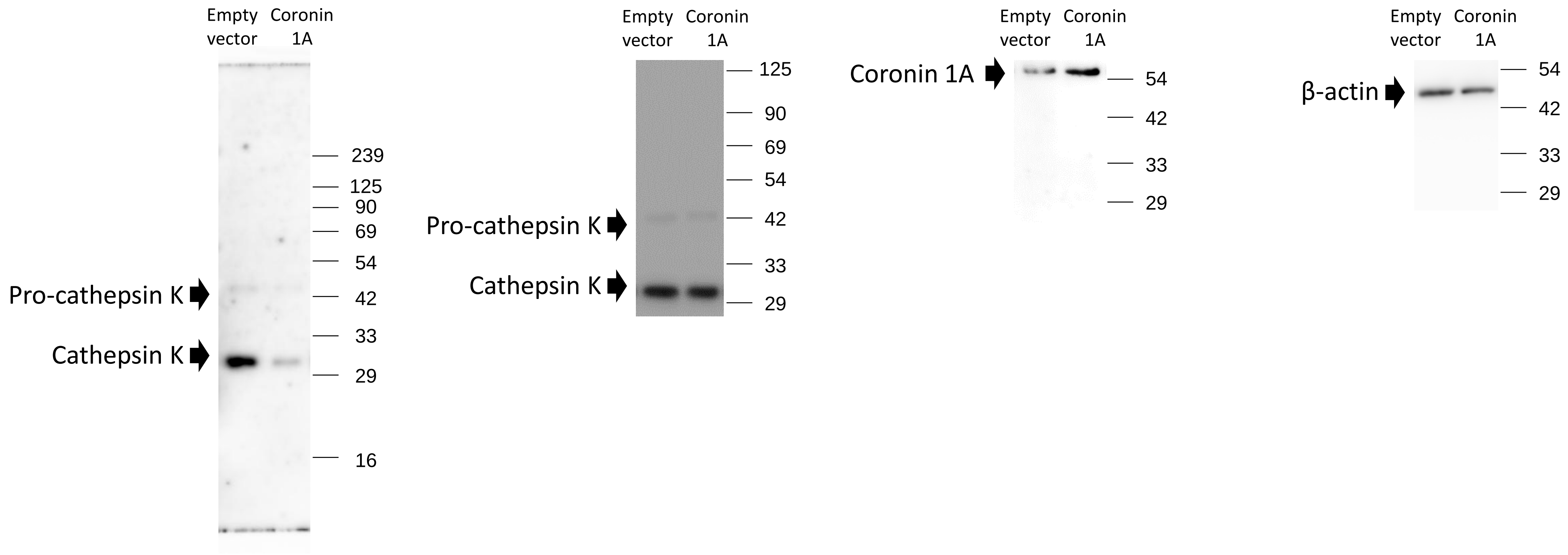

Fig. 3f

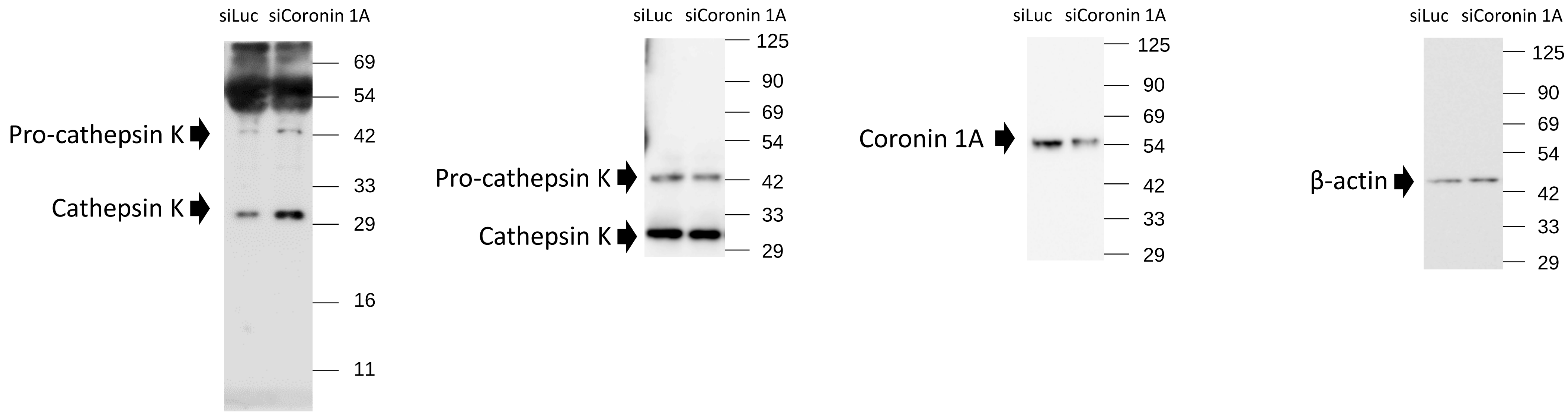

Supplementary Figure 10. Full-length immunoblot images in Figure 1, 3

All immunoblot images are full-length blot images of key blot data in figure 1, 3

Fig. 4a

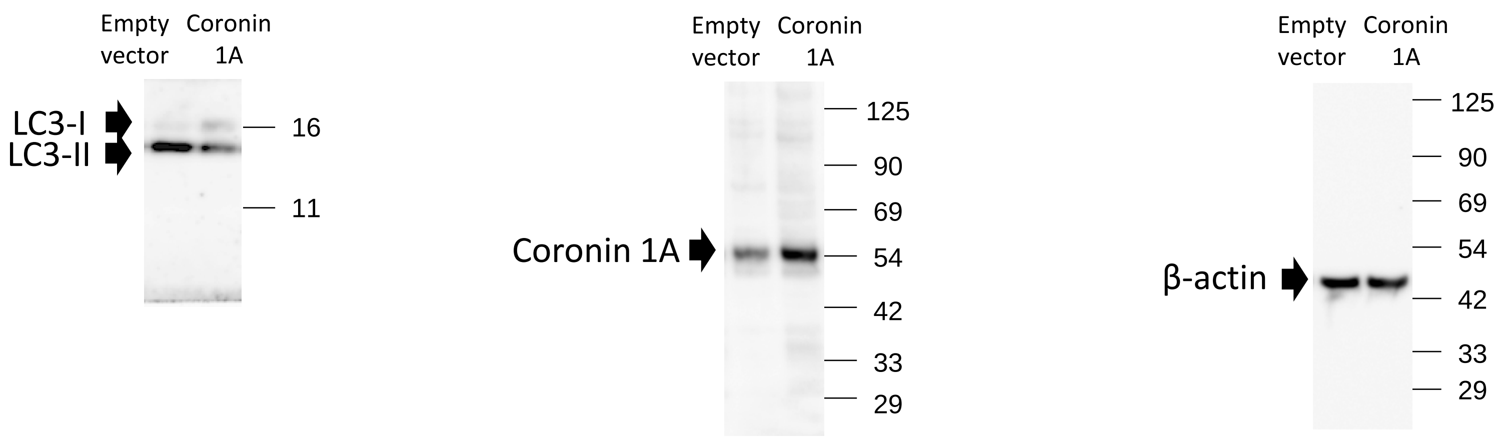

Fig. 4b

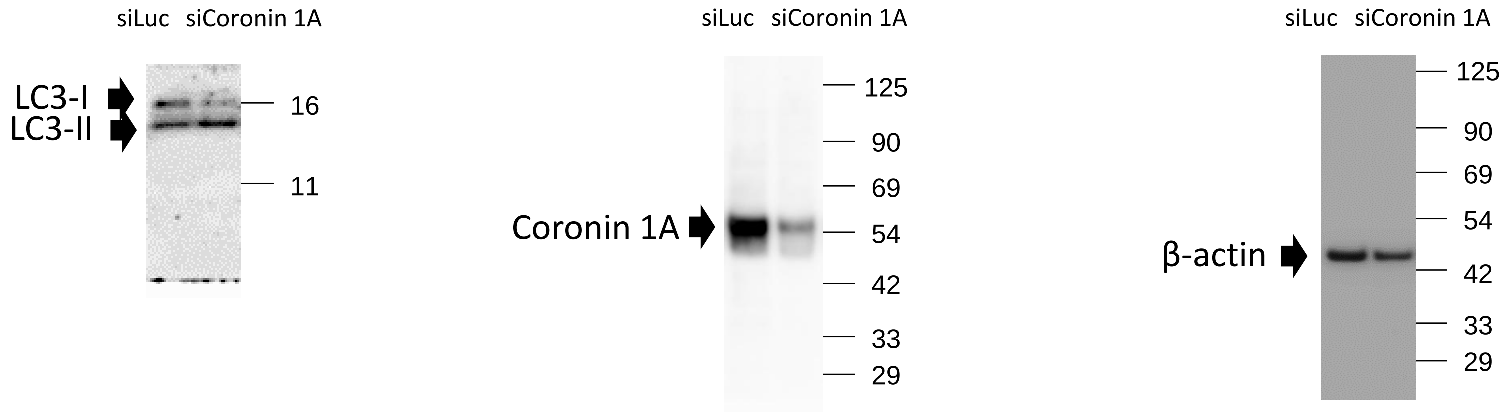

Fig. 5e

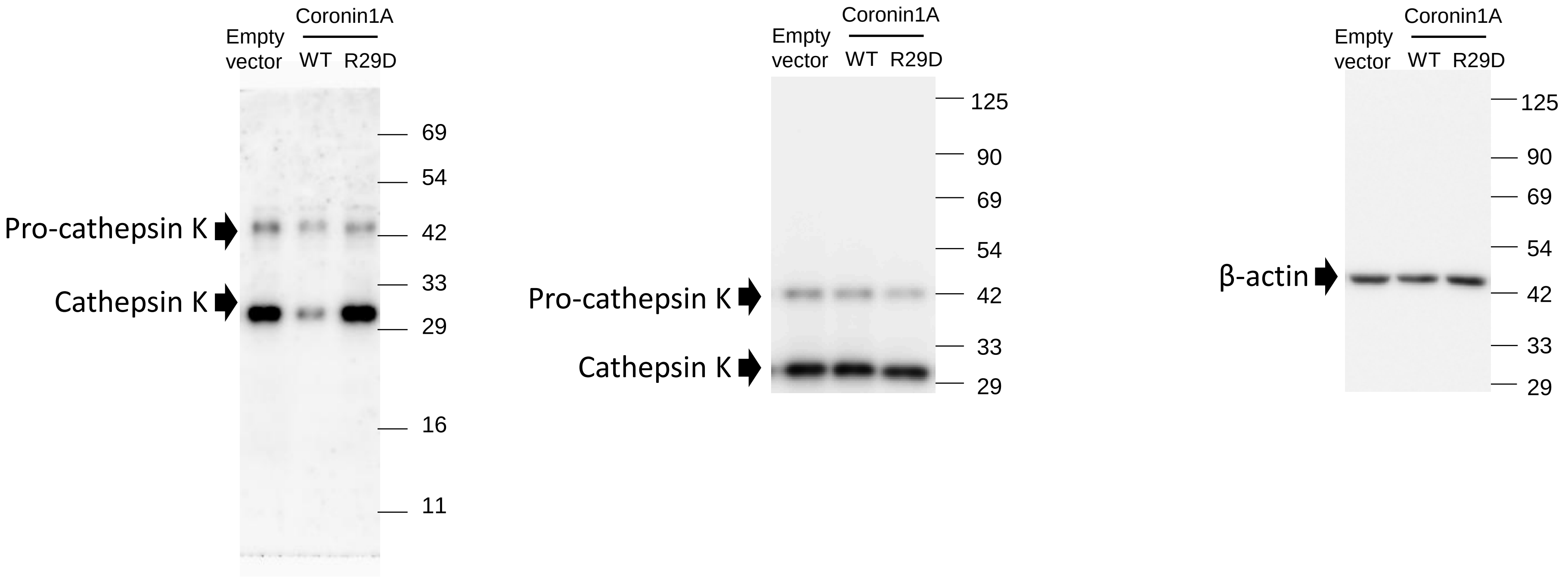

Fig. 5f

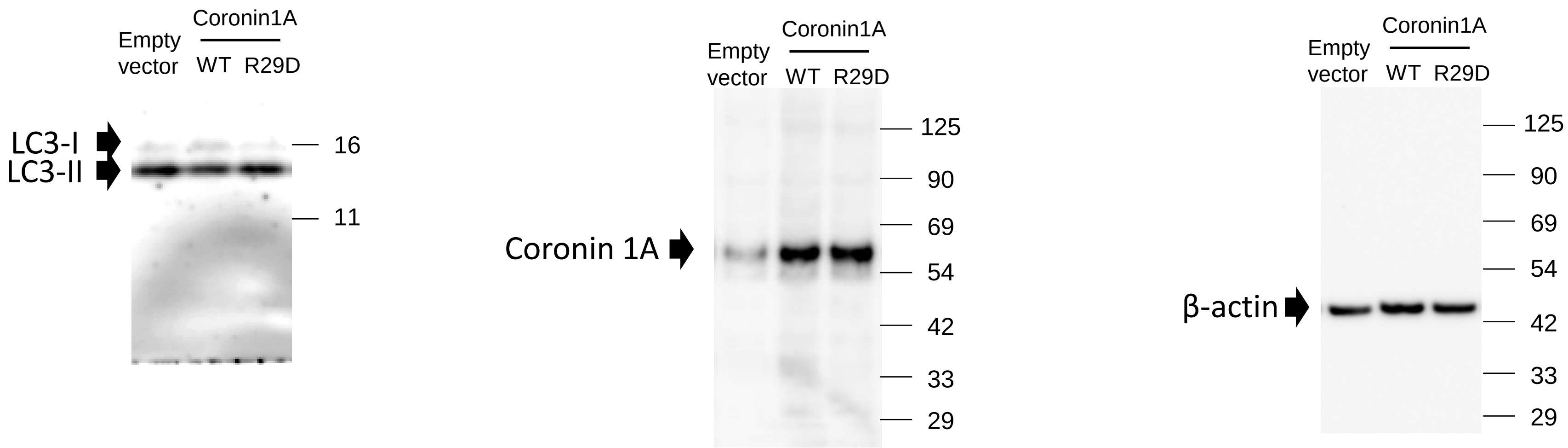

Supplementary Figure 11. Full-length immunoblot images in Figure 4, 5

All immunoblot images are full-length blot images of key blot data in figure 4, 5

Supplementary Fig. 1b, c

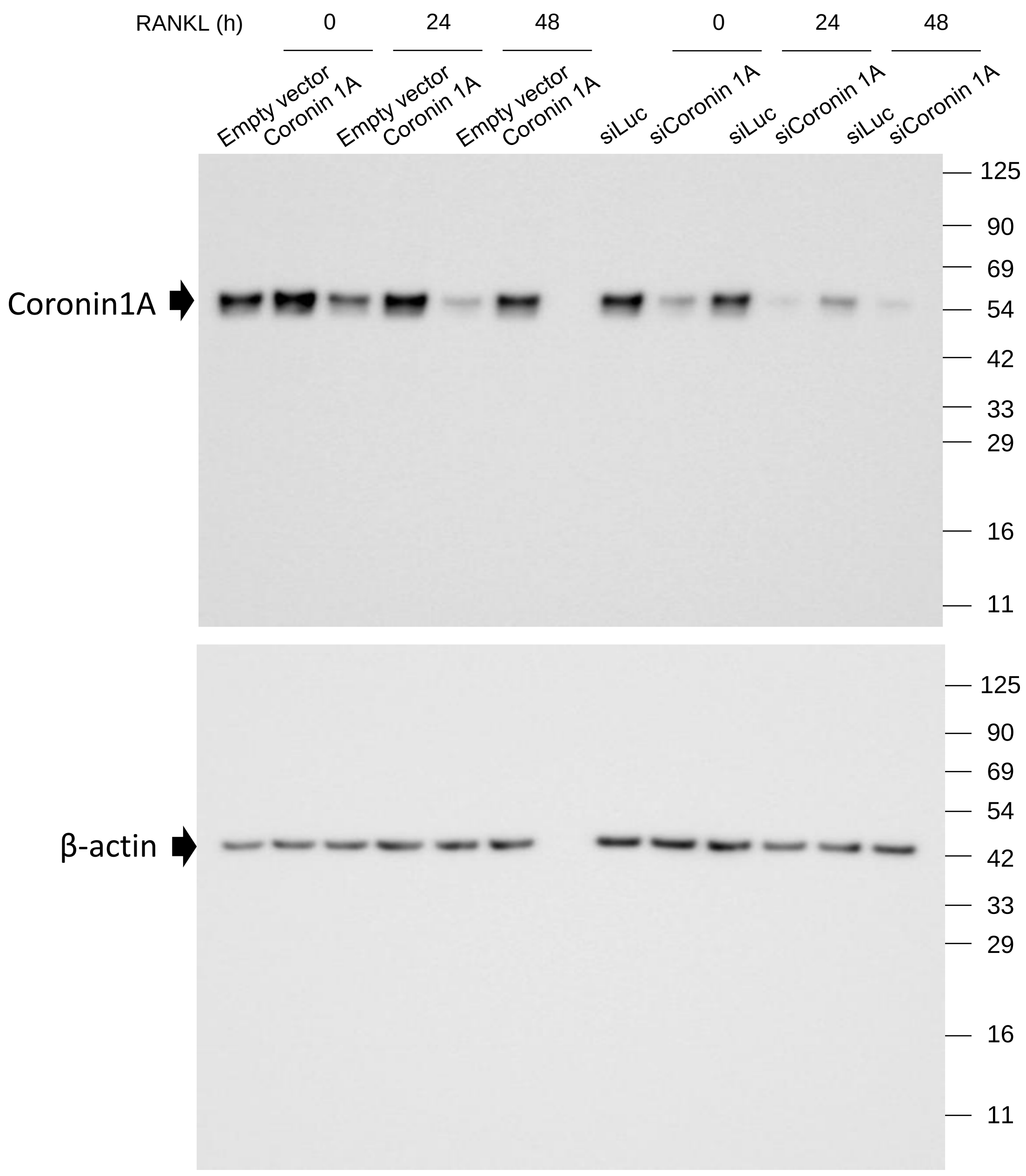

Supplementary Figure 12. Full-length immunoblot images in Supplementary figure 1
All immunoblot images are full-length blot images of key blot data in supplementary figure 1

Supplementary Fig. 3a

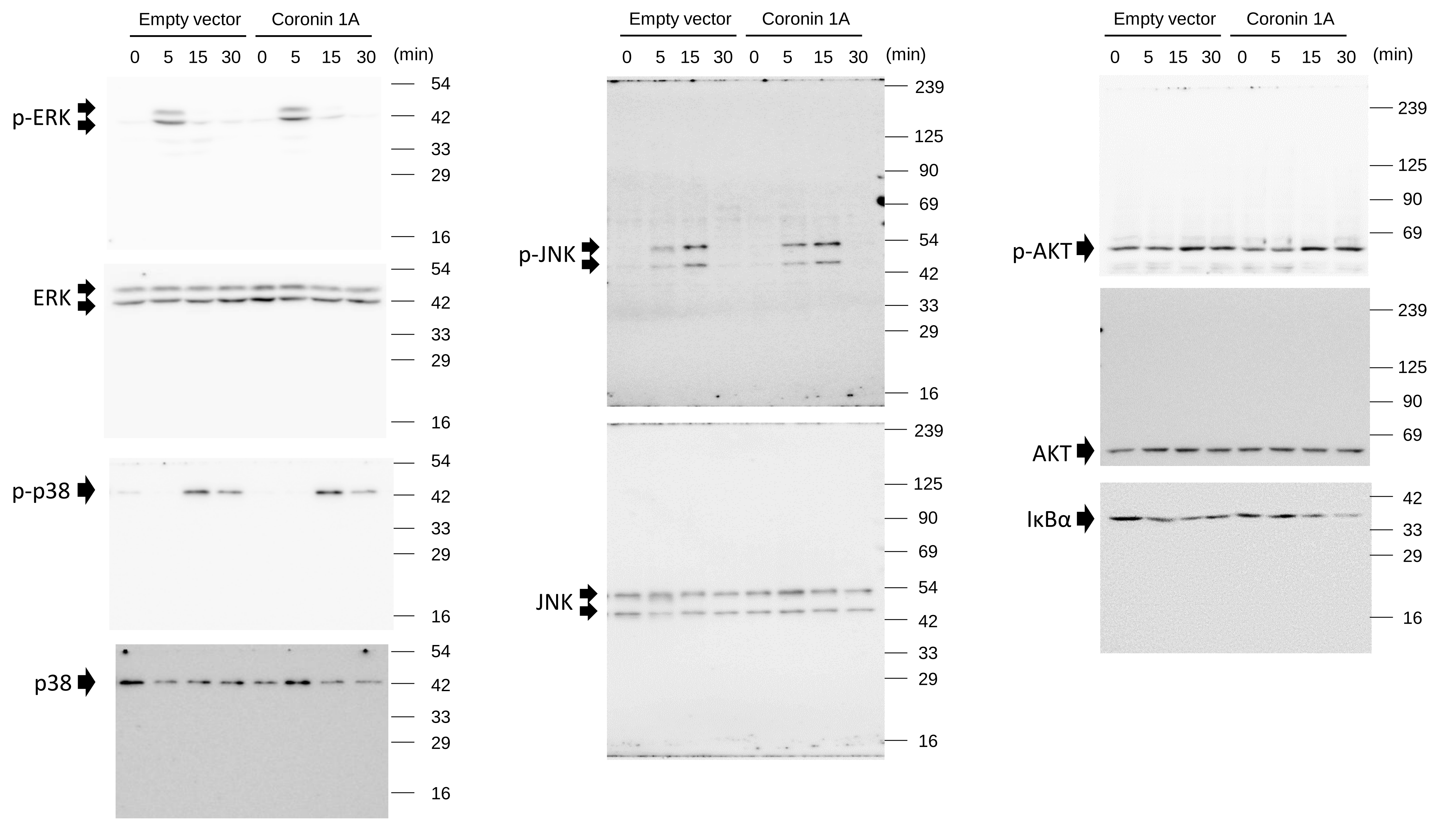

Supplementary Fig. 3b

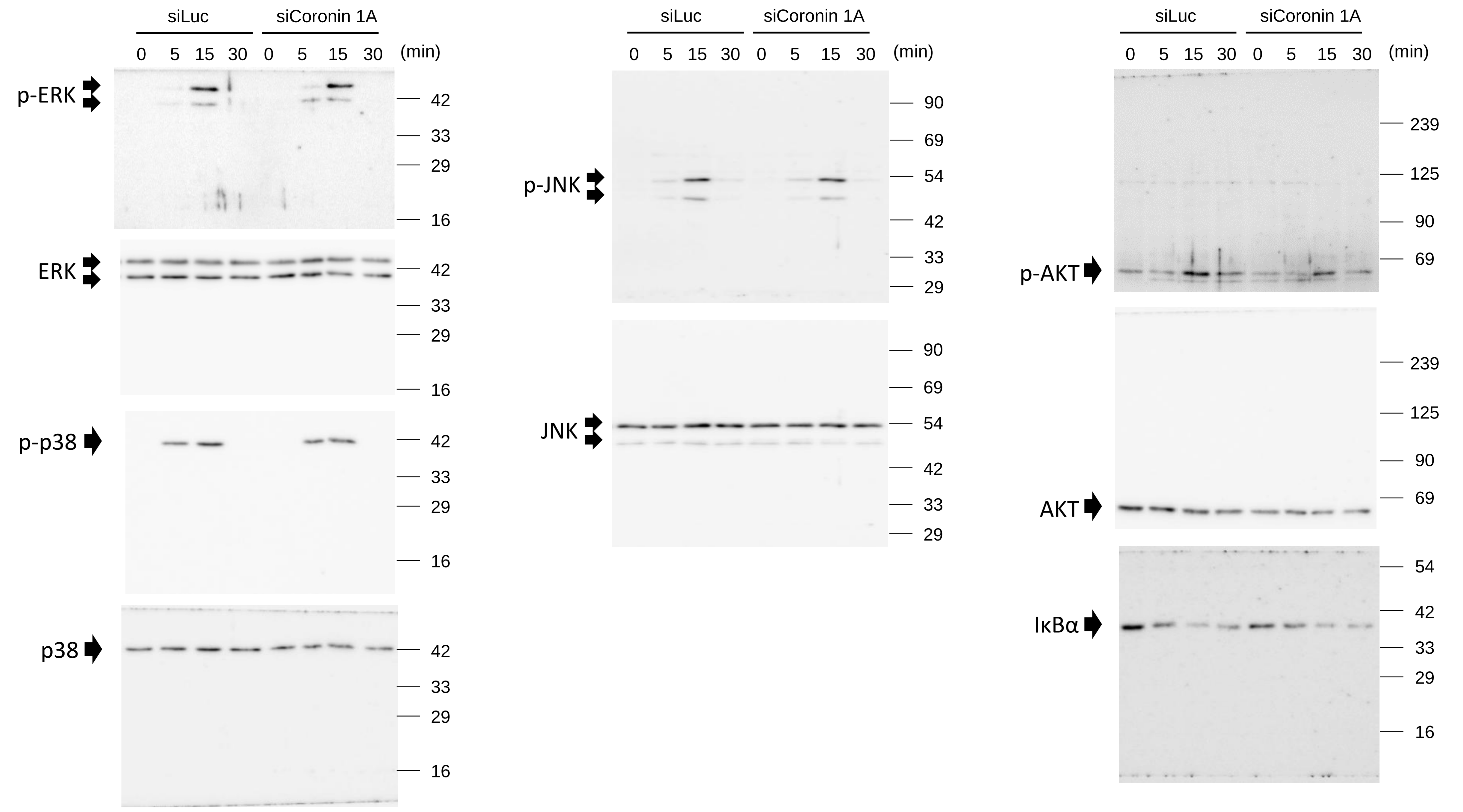

Supplementary Figure 13. Full-length immunoblot images in Supplementary figure 3

All immunoblot images are full-length blot images of key blot data in supplementary figure 3

Supplementary Fig. 7a

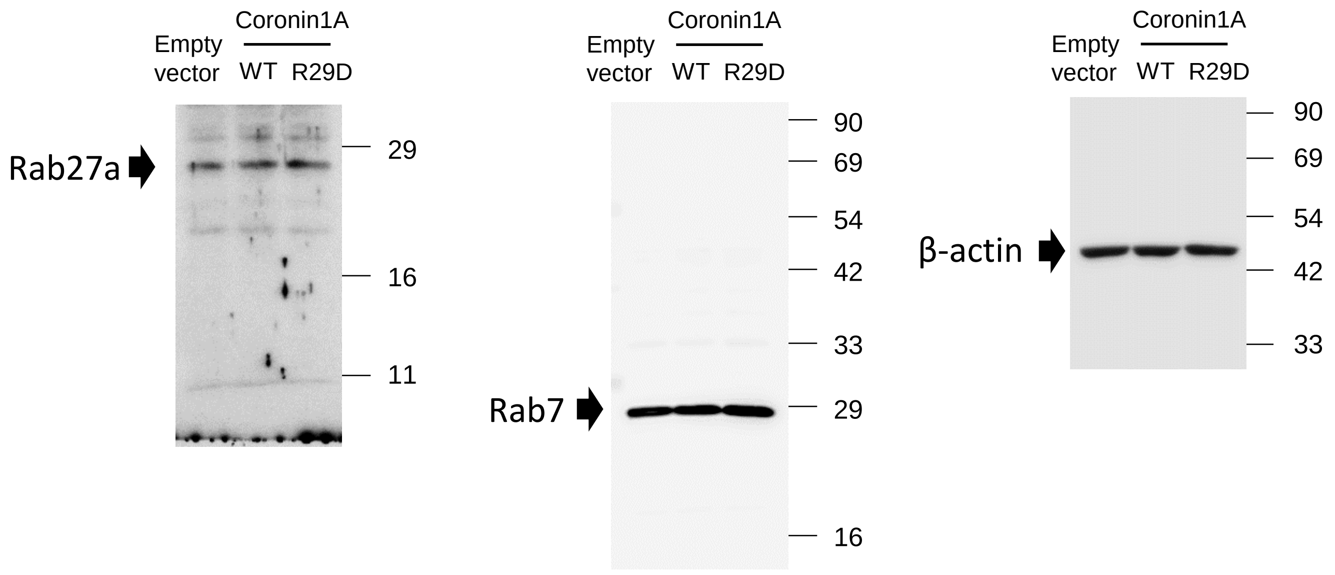

Supplementary Fig. 9c

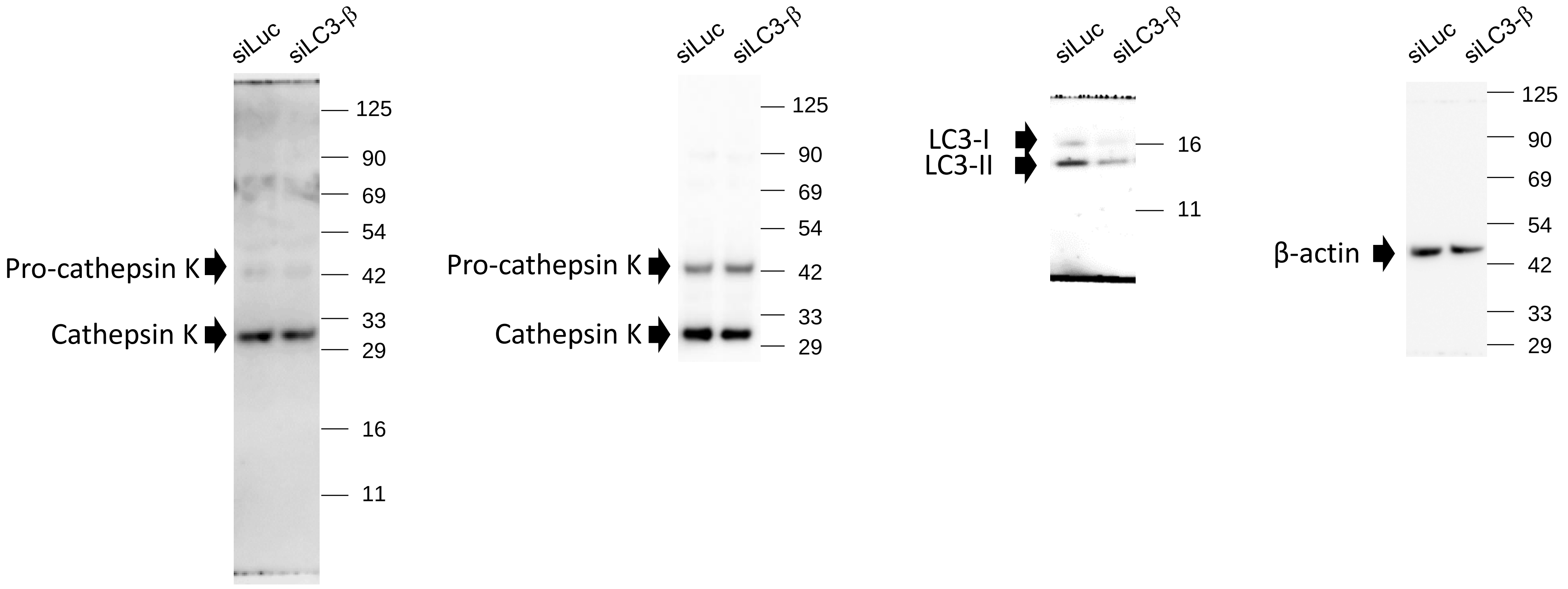

Supplementary Figure 14. Full-length immunoblot images in Supplementary figure 7, 9

All immunoblot images are full-length blot images of key blot data in supplementary figure 7, 9
